# Downregulated ferroptosis‐related gene SQLE facilitates temozolomide chemoresistance, and invasion and affects immune regulation in glioblastoma

**DOI:** 10.1111/cns.13945

**Published:** 2022-08-13

**Authors:** Lei Yao, Juanni Li, Xiaofang Zhang, Lei Zhou, Kuan Hu

**Affiliations:** ^1^ Department of Hepatobiliary Surgery Xiangya Hospital, Central South University Changsha China; ^2^ Department of Pathology Xiangya Hospital, Central South University Changsha China; ^3^ National Clinical Research Center for Geriatric Disorders Xiangya Hospital, Central South University Changsha China; ^4^ Departments of Burn and Plastic Ningxiang People's Hospital, Hunan University of Chinese Medicine Changsha China; ^5^ Department of Anesthesiology Third Xiangya Hospital of Central South University Changsha China

**Keywords:** chemoresistance, ferroptosis, GBM, SQLE, TMZ

## Abstract

Chemoresistance in patients with glioblastoma multiforme (GBM) is a common reason hindering the success of treatment. Recently, ferroptosis has been reported to be associated with chemoresistance in different types of cancer, while the role of ferroptosis‐related genes in GBM have not been fully elucidated. This study aimed to demonstrate the roles and mechanism of ferroptosis‐related genes in chemoresistance and metastasis of GBM. First, two candidate genes, squalene epoxidase (SQLE) and FANCD2, were identified to be associated with ferroptosis‐related chemoresistance in GBM from three temozolomide (TMZ) therapeutic datasets and one ferroptosis‐related gene dataset. Then, comprehensive bio‐informatics data from different databases testified that SQLE was significantly downregulated both in GBM tissue and cells and displayed a better prognosis in GBM. Clinical data identified lower expression of SQLE was significantly associated with WHO grade and 1p/19q codeletion. Moreover, through in vitro experiments, SQLE was confirmed to suppress ERK‐mediated TMZ chemoresistance and metastasis of GBM cells. The KEGG analysis of SQLE‐associated co‐expressed genes indicated SQLE was potentially involved in the cell cycle. Furthermore, SQLE was found to have the most significant correlations with tumor‐infiltrating lymphocytes and immunomodulators. These findings highlighted that SQLE could be a potential target and a biomarker for therapy and prognosis of patients with GBM.

## Graphical abstract

Chemoresistance in glioblastoma multiforme (GBM) is a common reason for tumor recurrence and ferroptosis has been reported to correlate with chemoresistance in cancers. Here, we identified a ferroptosis‐related gene SQLE that was significantly associated with the prognosis of patients with GBM. Moreover, SQLE was confirmed to suppress ERK‐mediated TMZ chemoresistance and metastasis of GBM cells. Our study proposed a novel target for the treatment of chemoresistant in GBM.

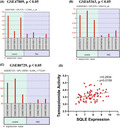

## 1 INTRODUCTION

Gliomas are the most prevalent type of central nervous system associated primary tumor,^1^ and are classed as four grades based on the WHO classification, including grades I, II, III, and IV.^2^ Despite satisfactory surgery and concurrent chemoradiotherapy, the median overall survival of patients with GBM was only 14.6 months.^3^, ^4^ Newly discovered biomarkers that predict improved survival include isocitrate dehydrogenase mutation and a glioma cytosine–phosphate–guanine island methylator phenotype.^5^ Moreover, 1p/19q co‐deletion is suggested as a beneficial prognostic factor for glioma patients.^6^ Furthermore, immunotherapy is gradually regarded as a particularly promising way of tumor therapy since can stimulate the immune system and attack tumor cells.^7^, ^8^ However, the significant changes in some critical regulators related to the immune response in GBM lead to tumor immune escape.^9^ Although several advances in the treatment for glioma, the prognosis is still very poor because of the tumor recurrence and chemoresistance for patients with GBM. Therefore, there is an urgent need to identify new drug targets and more specific therapies.

Ferroptosis is a form of non‐apoptotic, iron‐dependent, regulated cell death that is widely implicated in several pathological situations like drug resistance and brain injury.^10^ It is characterized by the failure of the glutathione‐dependent lipid peroxides defense network.^11^ Currently, studies have suggested that ferroptosis‐related chemoresistance has been reported in many types of malignancies,^12^, ^13^, ^14^ but their function in GBM therapy was not clear. Squalene epoxidase is a member of the flavoprotein monooxygenase family,^15^ which is the key rate‐limiting enzyme in cholesterol biosynthesis.^16^ What's more, following the lanosterol synthesis, cholesterol biosynthesis inhibition at different steps leads to the inhibition of cell death, cell differentiation induction, and cell cycle progression.^17^ Recently, human SQLE has attracted more and more attention as a bonafide oncogene and a target of tumor treatment.^17^, ^18^ As a ferroptosis regulator, SQLE was identified as a crucial cancer‐promoting gene in the breast cancer.^19^ Moreover, previous studies indicated that SQLE reduction disrupted the GSK3β/p53 complex, and helped colorectal tumor cells surmount constraints by evoking the epithelial‐mesenchymal transition (EMT) process required to produce tumor stem cells.^20^ Cancer stem cells possess the properties of self‐renewal and drug resistance and are closely linked to tumor metastasis and drug recurrence. Lnc‐030 can cooperate with PCBP2 to stabilize SQLE mRNA, and then activate PI3K/Akt signaling, which governs BCSC stemness.^21^ Although, the detailed role and mechanism of SQLE in the tumorigenesis and progression of GBM have not been explored.

This study investigated the detailed functions and mechanisms of ferroptosis‐associated genes for GBM chemotherapy. One ferroptosis‐related gene, SQLE, was identified to have an impact on chemotherapy response in GBM. Furthermore, lower SQLE expression was recognized in GBM and was correlated with poor prognosis. The expression levels of SQLE might be a positive correlation with TMZ activity in glioma cells. In addition, in vitro studies, we tested that SQLE was lowly expressed in TMZ‐resistant GBM cells and involved in ERK‐mediated TMZ resistance of GBM cells. Overexpression of SQLE could significantly inhibited the migration and invasion of GBM cells. Moreover, the KEGG analysis showed SQLE was potentially involved in the cell cycle. Furthermore, results of tumor‐immune system interactions and drug bank database (TISIDB) demonstrated that SQLE had the most significant correlations with tumor‐infiltrating lymphocytes and immunomodulators.

## 2 MATERIALS AND METHODS

### 2.1 Data acquisition and reanalysis using different bioinformatics tools

The functions of ferroptosis‐related chemoresistance of GBM were investigated using several public databases (Table S1). Three TMZ therapeutic transcriptome microarray datasets, GSE47809, GSE65363, and GSE80729,^22^ were obtained from the GEO database^23^ (Table S2). In GSE80729, two groups (U87 siCtrl, DMSO and U87 siCtrl, TMZ) were selected to analyze. Co‐differentially expressed genes (co‐DEGs) among these three TMZ‐associated datasets were recognized through a Venn diagram analysis. Next, the comprehensive ferroptosis‐related gene set was downloaded from a previous article^24^ (Table S3). Two ferroptosis‐related genes were identified between the co‐DEGs and the ferroptosis‐related gene set via a Venn diagram analysis.^25^ GlioVis^26^ was used to investigate the relationships between the significance of the two alternative genes and GBM prognoses.

The expression levels of SQLE in GBM were explored through the GlioVis, The Human Protein Atlas (THPA),^27^ Cancer Cell Line Encyclopedia (CELL),^28^ and OncoScape.^29^ The relationships between SQLE expression and clinicopathological factors of GBM were estimated using the Xiantao tool, which is an online tool including gene expression profiles from TCGA. The relationship between SQLE expression and MGMT promoter status was analyzed using cBioportal. Moreover, the aforementioned three TMZ‐associated datasets were applied to investigate the impact of GBM chemotherapy response on the expression of SQLE. The correlations between SQLE expression and drug sensitivity among GBM cell lines were gained from the CellMinerCDB.^30^ Besides, we obtained SQLE‐related coexpression molecular in GBM pathology via GlioVis, then a protein–protein interaction (PPI) was constructed via STRING.^31^ Next, Cytoscape software was used to accomplish detailed visualization.^32^ Moreover, GO functional enrichment analysis and KEGG pathway analysis was conducted by GSEA.^33^ The TISIDB database is an online integrated viewer for the analysis of interactions between tumors and the immune system.^34^ In our study, we investigated the correlation between SQLE expression and lymphocytes (TILs) and immunomodulators in GBM via TISIDB.

### 2.2 Cells and reagents

The TMZ‐resistant glioma cell lines (T98g‐R and U118MG‐R) and their parental cell lines (T98G and U118MG) were cultured as previously described.^35^, ^36^ All the cells were cultured in DMEM with 10%FBS. KO‐947 (Selleck Chemicals) was dissolved in DMSO, and the exposed concentration was 10 μM. Lipo3000 reagent was purchased from Invitrogen (Carlsbad, CA).

### 2.3 Colony formation assay

The T98G‐R and U118MG‐R cells were sufficiently trypsinized and suspended in a complete medium. Then, cells were seeded in 6‐well plates (500 cells/well) and cultured under 5% CO_2_ at 37°C. After two weeks, cell colonies were fixed with 4% paraformaldehyde (30 min), and stained with 0.1% crystal violet (20 min). Colonies with more than 50 cells will be counted.

### 2.4 Transwell assay and wound healing assay

For invasion assays, the upper chamber was placed into the 24‐well lower chamber. The upper membranes were coated with 40 μl of matrigel (Matrigel™ GFR Membrane Matrix, #356231, Corning, the USA) in advance. The cells were resuspended in FBS‐free medium and were seeded in the upper chamber (4 × 10^4^/chamber), medium with 20% FBS was added into the lower chamber. After 24 h, the cells were fixed (4% paraformaldehyde) and stained (0.1% crystal violet). Under the microscope, each group randomly selected 10 high‐power fields of view for counting and statistics.

For the wound‐healing assay, T98G‐R and U118MG‐R cells were seeded in 6‐well plates and cultured under 37°C and 5% CO_2_ for 24 h. When an adherent confluent monolayer formed, cell starvation was carried out on the experimental medium without FBS for 8 h to prevent cell proliferation. Next, a wound was made by scratching the monolayer cells with a sterile 10 μl pipette tip and washed with PBS. Subsequently, a fresh FBS‐free medium was added to the scratched T98G‐R and U118MG‐R cells. Photographs were taken through an inverted microscope at 0 and 12 h. Wound healing was determined as a percentage of wound confluence.

### 2.5 Protein extraction and immunoblotting

Protein extraction and immunoblotting were performed as we previously described.^36^ Primary antibodies used in these studies included SQLE (12544‐1‐AP, ProteinTech), ERK1/2 (#4695, CST), p‐ERK1/2 (#4370, CST), and β‐Tubulin (GB11017, Servicebio). Proteins were visualized with the enhanced chemiluminescence detection system according to the manufacturer's protocol (Bio‐Rad, Berkeley, CA, USA).

### 2.6 Plasmids and transient transfection

The SQLE overexpression plasmids (G0206627‐1) and blank plasmids (G0206627‐2) were constructed by Genepharma (Shanghai, China). The ERK overexpression plasmid was constructed by WZ Biosciences Inc (CH809152, Shandong, China). T98G‐R and U118MG‐R cells were transfected with these plasmids using Lipofectamine 3000 reagent (Invitrogen).

### 2.7 Statistical analyses

Statistical analyses were performed with SPSS 19.0 (IBM Analytics, USA) and GraphPad Prism 8 (San Diego California USA) software. Normality was analyzed using the Shapiro–Wilk test. Student's *t*‐test was applied to compare differential expression between two groups. The Kaplan–Meier analysis was used to analyze GBM prognosis. The Kruskal–Wallis rank test or the Mann–Whitney *U* test was used to analyze the associations between SQLE expression and clinicopathologic characteristics. Pearson's correlation coefficient was used to analyze the correlations between genes. All the experiments were conducted in triplicate with mean ± SD. *p < 0.05, **p < 0.01, and ***p < 0.001 were defined as statistically significant.

## 3 RESULTS

### 3.1 SQLE shows the significant prognostic value in GBM

**FIGURE 1:**
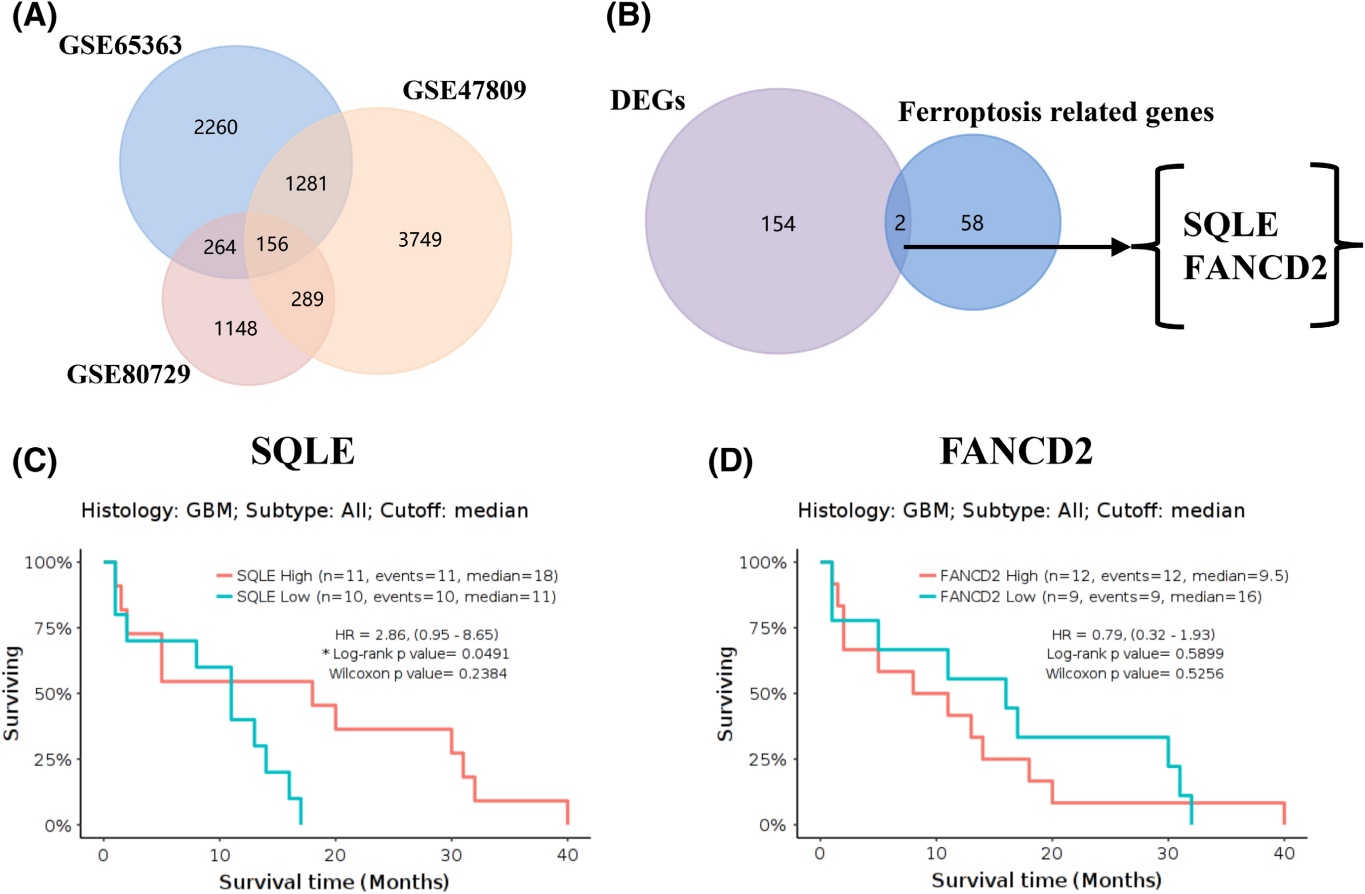
SQLE is significantly correlated with the prognosis of patients with GBM. (A) Venn diagrams showing 156 overlap genes from three GBM TMZ related therapeutic datasets. (B) Venn diagrams showing two overlap genes of 156 co‐DEGs and ferroptosis related gene set. (C, D) Overall survival curves for GBM patients on the basis of SQLE and FANCD2 expression (GlioVis).

Resistance to TMZ, the standard chemotherapy agent for glioma, poses a main challenge to GBM prognosis.^37^ We analyzed the gene expression profiles from three TMZ‐related therapeutic datasets (GSE47809, GSE65363, and GSE80729), to screen the co‐DEGs between the TMZ group and the control group. The screening thresholds was adjusted *p* < 0.05 and |logFC| > 2, we identified 5,475 genes in GSE47809, 3961 genes in GSE65363 and 1857 genes in GSE80729. Next, 156 genes were further identified to be significantly co‐differentially expressed among the aforementioned three datasets by the Venn analysis (Figure 1A, Table S4). These co‐DEGs identified from these three TMZ‐related therapeutic datasets were presumed to have some effect on the treatment response in GBM.

As a hallmark of cancer, ferroptosis has been proved to play a significant role in the occurrence and development of glioma.^38^ Next, we further explored the effects of ferroptosis on GBM treatment response. Two ferroptosis‐related gene, SQLE and FANCD2, were identified between the ferroptosis‐related gene set^24^ and the co‐DEGs of the aforementioned three datasets (Figure 1B). These two genes were presumed to have an impact on ferroptosis‐related drug resistance in GBM. Moreover, we analyzed the roles of SQLE and FANCD2 on GBM prognosis by GlioVis database, and found that low‐SQLE expression was significantly associated with poor prognosis, but FANCD2 did not show prognostic value in GBM (Figure 1C,D). According to the aforementioned results, SQLE, as the only molecular possess promising prognostic significance in GBM, was selected for further research.

### 3.2 SQLE is upregulated in GBM and impacts the sensitivity of TMZ treatment

**FIGURE 2:**
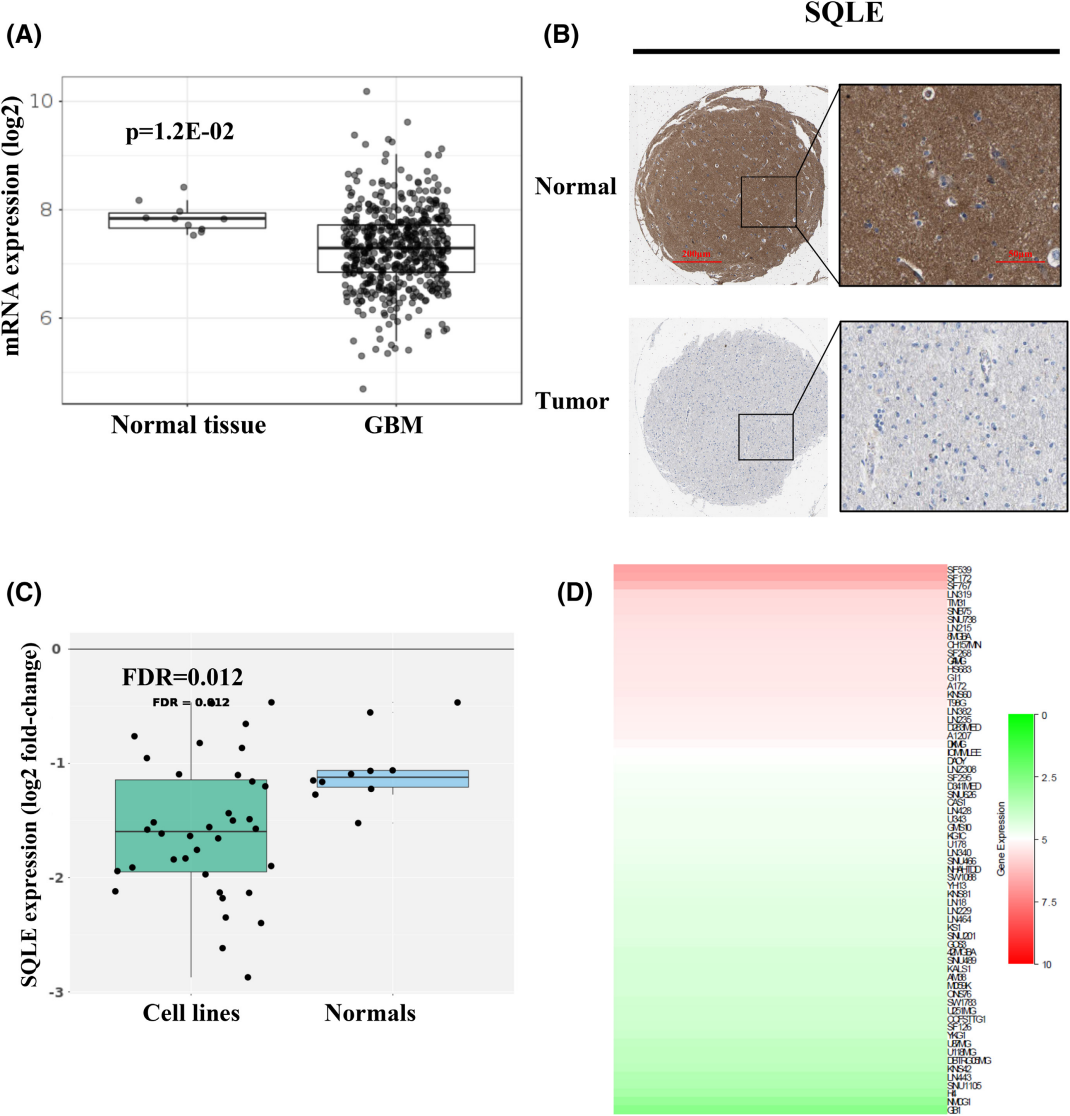
The expression levels of SQLE in GBM. (A) The mRNA expression of SQLE in GBM tissues (GlioVis databases). (B) the representative immunohistochemical images of SQLE in the paired tumor and adjacent normal tissues of GBM patients (The Human Protein Atlas). (C) The mRNA expression of SQLE in GBM cell lines (OncoScape). (D) The mRNA expression levels of SQLE in GBM cell lines (CCLE).

First, the mRNA and protein expression levels of SQLE in tissues were explored. We found that the mRNA expression of SQLE was lower in GBM tissues than that in cancer‐adjacent normal tissues (Figure 2A). Besides, the protein expression levels of SQLE were found to be obviously decreased in the GBM tissues by using THPA database (Figure 2B). In order to further verify the aforementioned conclusions, we further explored SQLE expression in glioma cell lines. As shown in Figure 2C,D, we found that the SQLE expression was relatively low in most cell lines (*p* = 0.012). Furthermore, we explored the correlation between SQLE expression and clinicopathological features in glioma, the results showed that SQLE expression was significantly associated with WHO grade and 1p/19q co‐deletion, and was not related to age, gender, IDH status, and MGMT methylation status (Table S5, Figure S1).

**FIGURE 3:**
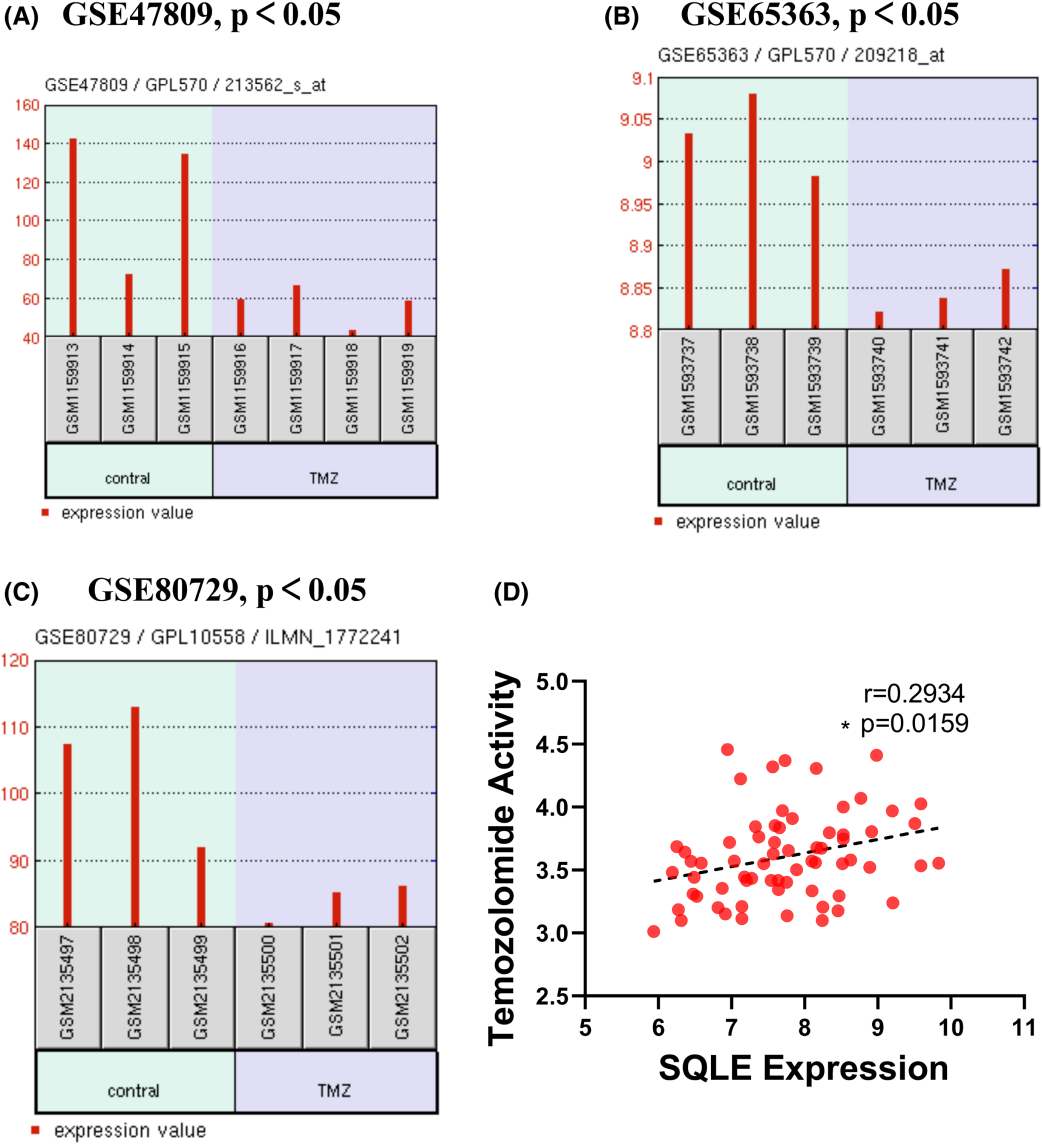
The relationship between TMZ treatment and SQLE expression in patients with GBM. (A–C) Three TMZ chemotherapy related microarray datasets were used to determine the effect of TMZ treatment on the expression level of SQLE. (D) SQLE expression was positive associated with TMZ activity in GBM cell lines (CellMinerCDB).

To further investigate the role of SQLE on the therapy outcomes in GBM, we eveluated the expression levels of SQLE in three TMZ related datasets including GSE65363, GSE80729, and GSE47809. The results exhibited that the TMZ treatment significantly decreased SQLE expression in patient tissues of GBM (Figure 3A–C). Besides, the expression levels of SQLE was positive correlation with TMZ activity in glioma cell lines (Figure 3D, Table S6). All those results demonstrated that SQLE expression was decreased in GBM, and significantly associated with WHO grade and 1p/19q co‐deletion, and may affected the sensitivity of patients with GBM to TMZ treatment.

### 3.3 SQLE is involved in TMZ resistance of GBM

**FIGURE 4:**
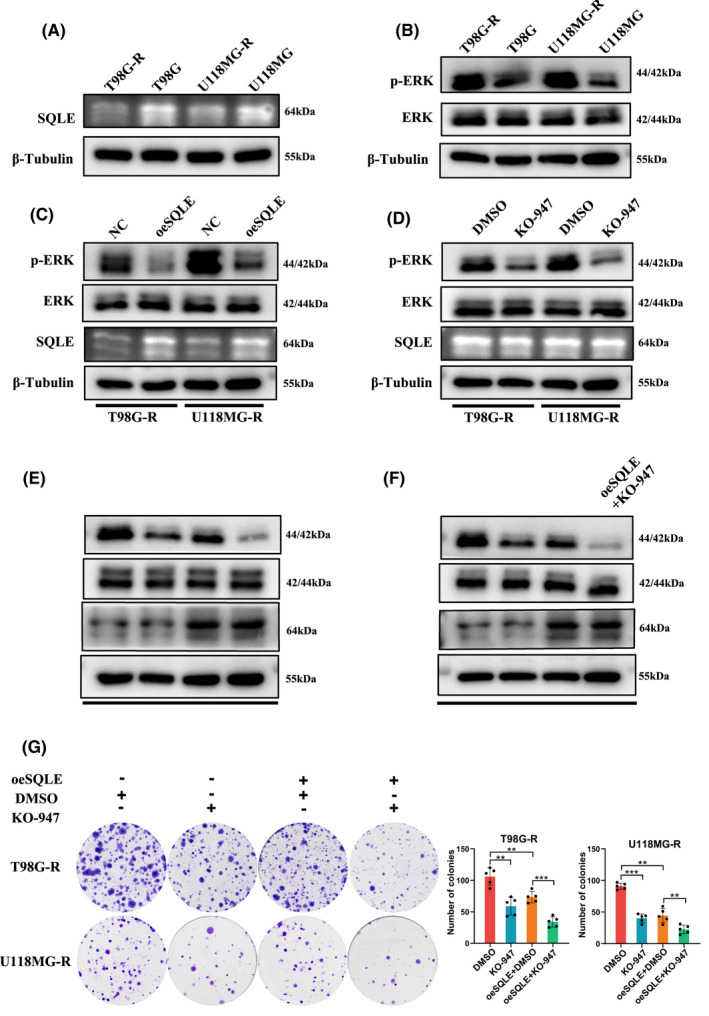
SQLE is involved in TMZ resistance of GBM. (A, B) The protein expression of SQLE, p‐ERK, and ERK between T98G‐R/U118MG‐R and T98G/U118MG. (C) Western Blot for SQLE, p‐ERK, and ERK in T98G‐R and U118MG‐R cells transfected with SQLE plasmid and control plasmid. (D) Western Blot for SQLE, p‐ERK, and ERK in T98G‐R and U118MG‐R cells transfected with KO‐947 and DMSO. (E, F) Western Blot for SQLE, p‐ERK, and ERK in T98G‐R and U118MG‐R cells transfected with DMSO, KO‐947, SQLE plasmid, and SQLE plasmid plus KO‐947. (G) Representative images and quantification of the Colony Formation assay in T98G‐R and U118MG‐R cells transfected with DMSO, KO‐947, SQLE plasmid, and SQLE plasmid plus KO‐947.

We further verify the inhibitory effect of SQLE on TMZ related chemoresistance in GBM. First, we detected the expression levels of SQLE between TMZ‐resistant glioma cells (T98G‐R and U118MG‐R cells) and their parental cells (T98G and U118MG cells). The results showed that SQLE expression decreased significantly in TMZ‐resistant glioma cell lines (Figure 4A), suggested that SQLE may play a crucial role in TMZ‐related chemoresistance in GBM.

Researches have shown that MAPK/ERK signaling pathway plays a pivotal role in the development and chemoresistance of GBM.^39^ Therefore, we further explored whether the effect of SQLE on TMZ chemoresistance of GBM was related to ERK activity. Our results showed that the expression of p‐ERK was significantly higher in TMZ‐resistant glioma cells, whereas total ERK expression has no change (Figure 4B). Moreover, overexpression of SQLE obviously suppressed phosphorylation of ERK in GBM, but down‐regulation of the phosphorylation of ERK with KO‐947, a tested ERK inhibitor, has no influence on SQLE expression, suggesting that SQLE may inhibit chemoresistance in GBM via MARK/ERK pathway by down‐regulated the phosphorylation of ERK (Figure 4C,D). Furthermore, cells were transfected with both SQLE overexpression plasmid and KO‐947, and the results showed that the inhibitory effect on phosphorylation of ERK was obviously increased by combinational treatment (Figure 4E,F). The colony formation assays were further performed, and the results showed that overexpression of SQLE or KO‐947 treatment decelerated T98G‐R and U118MG‐R cells colony formation, respectively. Interestingly, when overexpression of SQLE and treated KO‐947 simultaneously, the proliferation of GBM cell lines were decelerated the most (Figure 4G). Meanwhile, we overexpressed ERK after SQLE overexpression, and found that ERK overexpression could partially recover the decreased expression of p‐ERK and the decreased ability of cell proliferation caused by SQLE overexpression (Figure S2A–C). These results aforementioned were indicating that SQLE was involved in ERK‐mediated temozolomide resistance in GBM.

### 3.4 SQLE inhibits the migration and invasion ability of GBM

**FIGURE 5:**
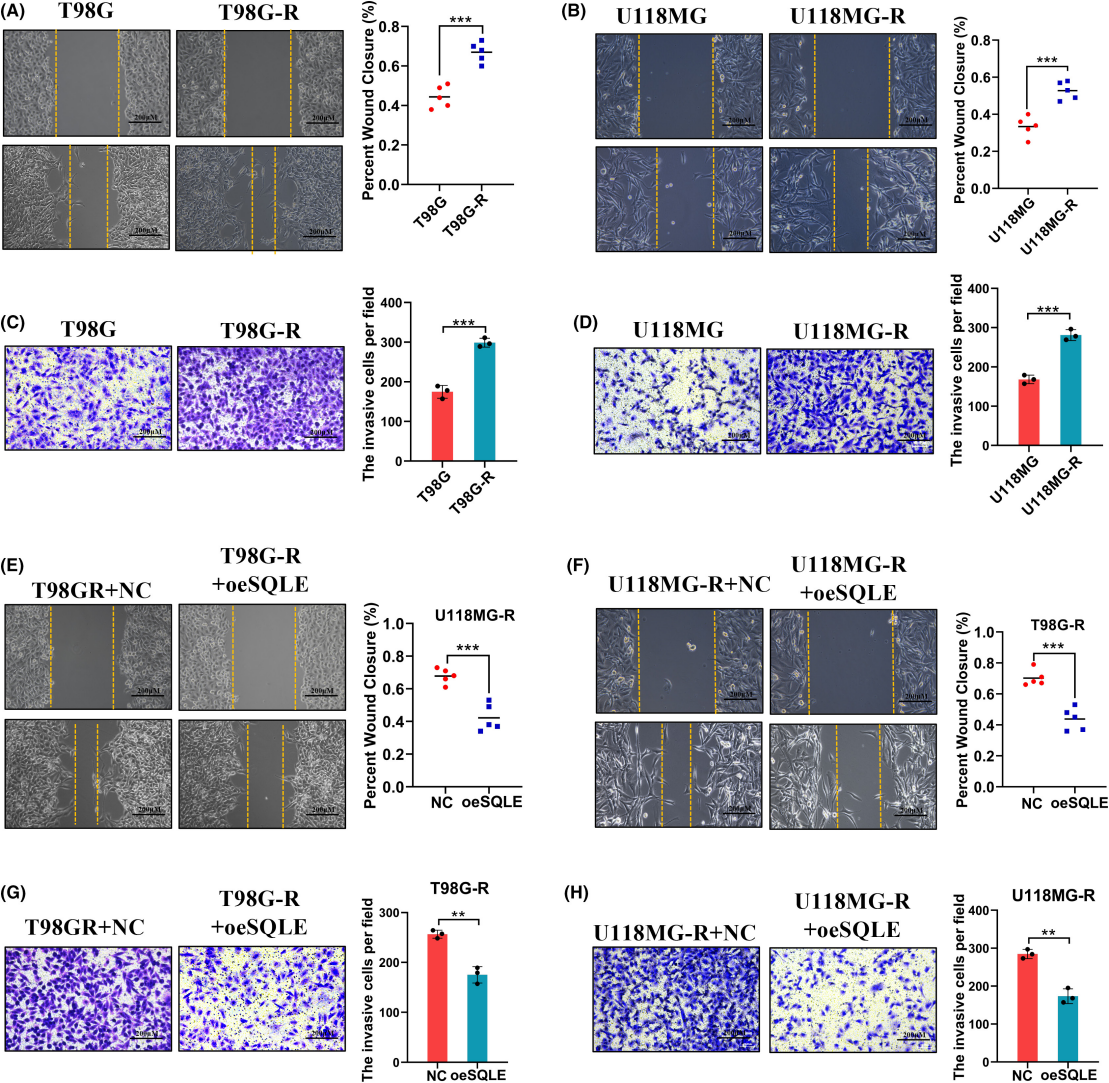
SQLE inhibits the migration and invasion ability of GBM. (A, B) Wound healing assay between T98G‐R/U118MG‐R and T98G/U118MG. (C, D) Transwell invasion assay between T98G‐R/U118MG‐R and T98G/U118MG. (E, F) Wound healing assay for T98G‐R and U118MG‐R cells treated with SQLE plasmid and control plasmid. (G, H) Transwell invasion assay for T98G‐R and U118MG‐R cells treated with SQLE plasmid and control plasmid.

Several studies demonstrated that ERK was involved in the invasion and metastasis of the GBM,^40^ we further explored whether the regulation of SQLE on ERK pathway affect the invasion and migration capacity of GBM cells. First, we found that the migration and invasion ability of TMZ‐resistant GBM cell lines were much stronger than their parental cell lines by using the wound‐healing assays and transwell assays (Figure 5A–D). Then, we overexpressed SQLE in two TMZ‐resistant GBM cell lines, and the results showed that the migration and invasion ability was significantly inhibited in these cells with SQLE overexpression, suggesting that SQLE play a critical role in the migration and invasion of GBM (Figure 5E–H).

**FIGURE 6:**
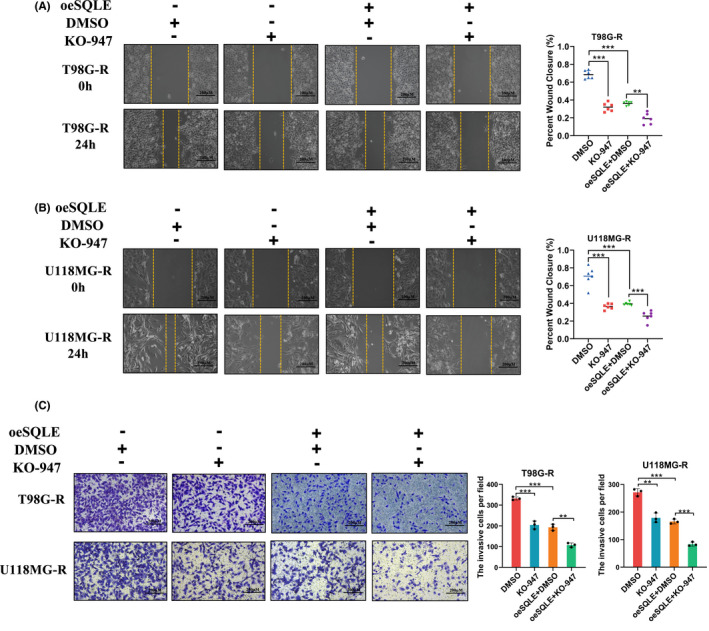
SQLE inhibits the migration and invasion of GBM via ERK pathway. (A, B) Wound healing assay for T98G‐R and U118MG‐R cells treated with DMSO, KO‐947, SQLE plasmid, and SQLE plasmid plus KO‐947. (C) Transwell invasion assay for T98G‐R and U118MG‐R cells treated with DMSO, KO‐947, SQLE plasmid, and SQLE plasmid plus KO‐947.

We further explored whether SQLE affects the migration and invasion ability of GBM cells through ERK pathway. We found that compared with either of the individual treatment groups, the migration and invasion ability of TMZ‐resistant GBM cells transfected with both SQLE overexpression plasmid and KO947 were suppressed most (Figure 6). Besides, we also overexpressed ERK after SQLE overexpression, the results showed that compared with SQLE overexpression group, ERK overexpression after SQLE overexpression could partially recover the decreased ability of cell migration and invasion caused by SQLE overexpression (Figure S2D–F). All those results indicated that SQLE negatively regulated p‐ERK and inhibited the metastatic ability of GBM cells in vitro.

### 3.5 Functional enrichment analysis of SQLE related co‐expressed genes

**FIGURE 7:**
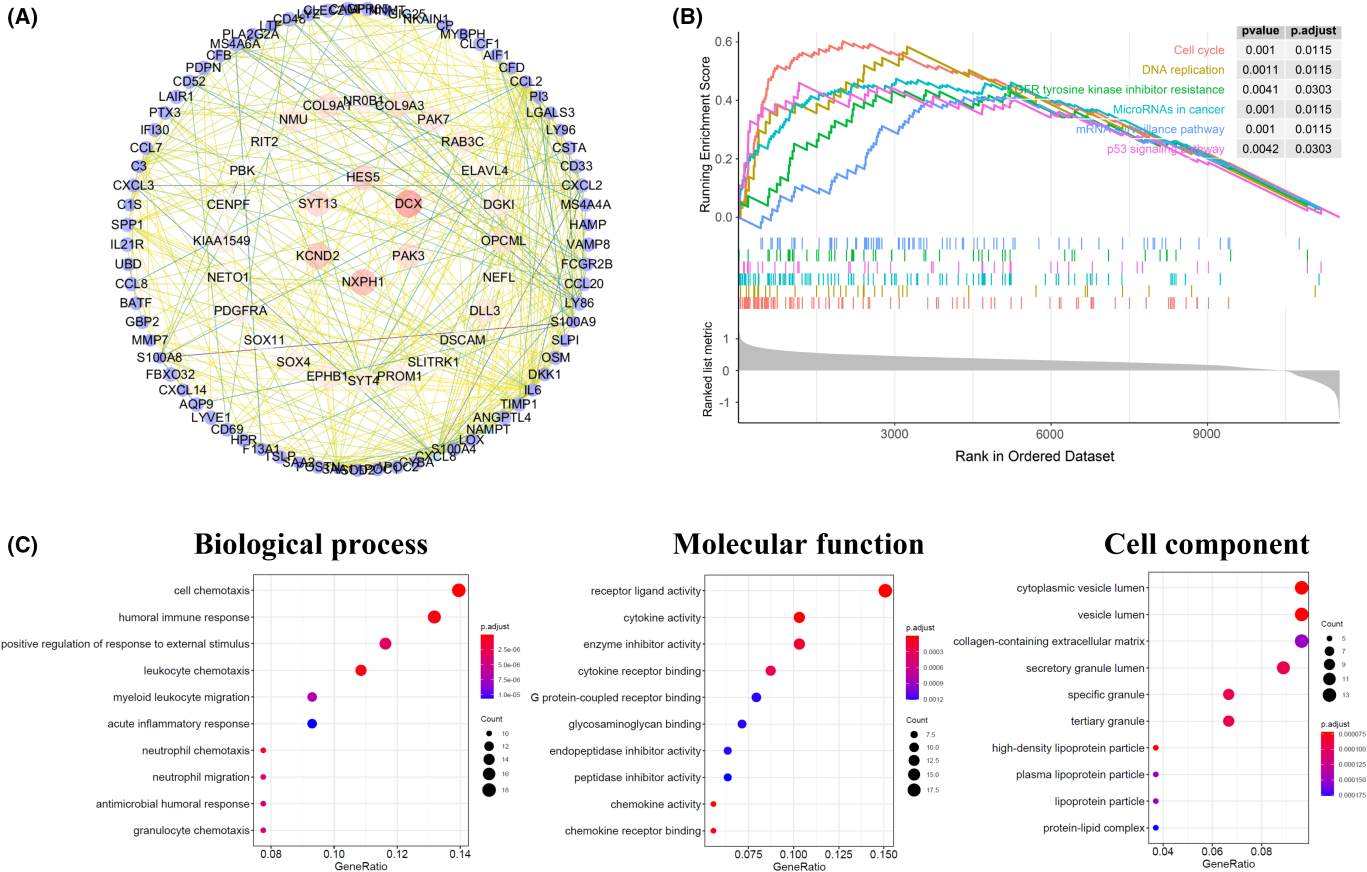
Functional enrichment analysis of SQLE‐associated co‐expression genes. (A) The PPI network of SQLE‐related co‐expression genes (STRING and Cytoscape). (B) The KEGG pathways associated with the SQLE co‐expression genes (GSEA). (C) The Biological Processes, Molecular function, and Cell component associated with the SQLE co‐expression genes (GSEA).

To further explore the potential capacity of SQLE in the tumorgenesis and development of GBM, we accomplished functional enrichment annotation analysis of its co‐expressed genes. First, we screened DEGs that interact with SQLE from TCGA–GBM datastes through GlioVis database. There were 150 co‐expression genes and their |logFC| ≥ 1 and *p* < 0.01 (Figure S3, Table S7). Then, a PPI network of these moleculars that co‐expressed with SQLE was created, and it was found that DCX, NXPH1, KCND2, SYT13, HES5, and PAK3 were mainly related to the modulation and function of the SQLE differentially expressed in GBM (Figure 7A). Moreover, GO functional enrichment analysis and KEGG pathway analysis was performed (GSEA). The results exhibited these co‐expressed genes were rich in functions in multiple pathways, which mainly involved the cell cycle (Figure 7B,C).

### 3.6 Regulation of immune molecules by SQLE

Immune cell levels were correlated with tumorigenesis and progression in many types of cancer.^41^ In recent years, more and more evidences have exhibited tumor microenvironments and immune infiltration play a significant role in tumorgenerisis and chemoresistance.^42^ Therefore, we explored the association between the expression of SQLE and lymphocytes and immunomodulators in the TISIDB database. The expression of SQLE in patients with GBM was negatively correlated with tumor‐infiltrating lymphocytes (TILs), and the lymphocytes that exhibited the most significant associations included mast cells (Mast, Spearman: roh = −0.472, *p* = 1.97e−10), monocyte cells (Monocyte, Spearman: roh = −0.441, *p* = 4.14e−09), myeloid‐derived suppressor cells (MDSCs, Spearman: roh = −0.431, *p* = 9.39e−09), and activated dendritic cells (Act_DC, Spearman: roh = −0.428, *p* = 1.25e−08) (Figure S4A,B). Furthermore, the expression of SQLE in patients with GBM was negatively correlated with immunomodulators including immunoinhibitors, immunostimulators, and major histocompatibility complex (MHC) molecules. The immunostimulator that exhibited the most momentous associations contained TNFSF13 (Spearman: roh = −0.427, *p* = 1.33e−08), CD86 (Spearman: roh = −0.4415, *p* = 3.61e−08), C10orf54 (Spearman: roh = −0.376, *p* = 7.36e−07), and CD48 (Spearman: roh = −0.428, *p* = 1.25e−08) (Figure S4C,D); those immunoinhibitors exhibiting the most significant associations contained IL10 (Spearman: roh = −0.469, *p* = 2.84e−10), CD244 (Spearman: roh = −0.461, *p* = 6.39e−08), HAVCR2 (Spearman: roh = −0.436, *p* = 6.17e−09), and PDCD1LG2 (Spearman: roh = −0.342, *p* = 7.35e−06) (Figure S4E,F); and the MHC molecules that exhibited the most significant associations contained HLA–DMB (Spearman: roh = −0.463, *p* = 5.02e−10), HLA–DRA (Spearman: roh = −0.414, *p* = 3.87e−08), HLA–DMA (Spearman: roh = −0.406, *p* = 7.48e−08), and HLA–DOA (Spearman: roh = −0.351, *p* = 4.14e−06) (Figure S4G,H). Based on the significant correlation between SQLE and tumor‐infiltrating lymphocytes, immunosuppressants, immunostimulants, and MHC molecules in GBM, we speculate that it may have a more significant impact on the immune infiltrating in GBM.

## 4 DISCUSSION

There is an urgency to find effective biomarkers and improve treatment strategies based on the fact that patients with GBM suffer from poor prognosis and chemoresistence. A recent study showed that the ferroptosis gene signature could predict immunotherapy and prognosis in patients with glioma, indicating that ferroptosis plays an important role in the progression of glioma.^43^ In our research, we aimed to explore crucial and novel biomarkers implicated in the development of ferroptosis‐related drug resistance of GBM. Intriguingly, SQLE and FANCD2 were identified by screening co‐DEGs between one ferroptosis‐related gene set and three TMZ‐related chemoresistance databases. But only SQLE showed a promising prognosis significance in GBM. As a candidate, SQLE was also found to be down‐regulated in GBM and affect the treatment results in GBM. Therefore, its biological processes and its correlations with immune infiltration will be intensively investigated.

SQLE is the key rate‐limiting enzyme in cholesterol biosynthesis and a target of fungicides and increasing interest in human health and disease.^16^ Previous studies revealed that, as a ferroptosis regulator, SQLE was associated with the proliferation and metastasis of breast cancer.^19^ Tumor‐related genes usually show two sides, inhibiting or promoting cancer. SQLE also acts either as a tumor suppressor or a tumor activator according to different cell types or cellular localization.^44^ Polycarpou‐Schwarz et al. proved that downregulation of CASIMO1 reduced the phosphorylation of ERK and the protein abundance of SQLE, and inhibited the metastasis of breast cancer cells.^45^ Similarly, lnc030 cooperates with PCBP2 could stabilize the mRNA of SQLE to upregulate cholesterol synthesis, thus actives PI3K/Akt signaling to control BCSC stemness.^21^ Contrarily, Mahoney CE et al. demonstrated that as a tumor suppressor gene, the sensitivity to SQLE inhibition results mainly from the specific and toxic accumulation of the SQLE substrate, squalene.^18^ However, the detailed roles of SQLE in GBM were still not clear. In our study, we found that SQLE expression decreased in GBM, and significantly correlated with low‐tumor grade and 1p19q co‐deletion. Furthermore, we first demonstrated that SQLE was involved in ERK‐mediated TMZ resistance in GBM.

GBM is the most common type of grade IV gliomas.^46^ Although many tumor treatment have been improved in the past few decades, few effective drugs have been approved by FDA for GBM therapy because of the blood–brain barrier.^47^ Recent studies revealed that immune checkpoint inhibitors appear to be an effective anticancer strategy in glioma immunotherapy.^9^ More and more evidences have indicated that immune infiltration play a significant role in tumorigenesis and chemoresistance.^48^ In this study, we demonstrated that SQLE had the most significant correlations with tumor‐infiltrating lymphocytes (such as Mast, Monocyte, MDSC, and Act_DC), immunostimulators (such as TNFSF13, CD86, C10orf54, and CD48), immunoinhibitors (such as IL10, CD244, HAVCR2, and PDCD1LG2), and MHC molecules (such as HLA–DMB, HLA–DRA, HLA–DMA, and HLA–DOA). Dendritic cell vaccine (DCV) is a kind of vaccine composed of antigen‐presenting cells (APCs), which can effectively induce immune responses.^49^, ^50^ Cytokines were reported to play a critical role in regulating the activity of cancer cells and immune cells in GBM, mainly providing immunosuppressive role.^51^ In glioma cells, IL‐10 receptor activates JAK‐STAT3 pathway, which, in turn, regulates the proliferation and metastasis of tumor cells.^52^ Tumor‐associated macrophages (TAMs) are generally considered to be macrophages that specifically aggregate in the microenvironment around tumors and can promote tumor progression.^53^ Previous studies confirmed that there were more TAMs in high‐grade gliomas than in low‐grade gliomas using immunohistochemical staining.^54^ PD‐L1, also named CD274, is an immune checkpoint molecule interrelated to programmed cell death. PDL1/PD‐1 axis was reported to be related with Treg expansion inhibition and immunosuppression prevention in GBM.^55^ In brief, these outcomes suggest that SQLE, which is related to the aforementioned immune infiltrating molecules, plays a significant role in immune infiltrating in GBM and may serve as a promising immunotherapeutic target in GBM.

Nevertheless, our research still has some limitations. The expression and prognosis data of SQLE were mainly obtained from TCGA, which mainly included white and black, which made our conclusions suitable for limited ethnic populations. Besides, several genes are involved in ferroptosis related signaling pathways, such as GPX4, Nrf2, SOD, MDA, and so on.^56^ However, our research only explored the role of ERK. In future studies, we will further explore the role of other molecules that involved in ferroptosis‐related pathway in the progression of glioma.

To sum up, this is the first study that SQLE is associated with the ferroptosis‐related chemoresistance in GBM and the low expression of SQLE was related with poor prognosis and low‐immune infiltration. Furthermore, SQLE was related to ERK‐mediated TMZ resistance of GBM cells. Overexpression of SQLE could inhibited the migration and invasion ability of GBM cells. Therefore, SQLE could be used as a potential therapeutic target and as a promising prognosis biomarker for patients with GBM.

## AUTHOR CONTRIBUTIONS

All the authors made substantive intellectual contributions to this study to qualify as authors. Kuan Hu and Juanni Li conceived the design of the study. Lei Yao and Xiaofang Zhang performed the study, collected the data and contributed to the design of the study. Lei Zhou and Lei Yao edited the manuscript. All the authors read and approved the final manuscript.

## FUNDING INFORMATION

This study is supported by grants from the National Natural Science Foundation of China (82102743, 82103300), Outstanding Postdoctoral Innovative Talents Foundation (2021RC2022), Youth Science Foundation of Xiangya Hospital (2020Q07)

## CONFLICT OF INTEREST

The authors declare no conflict of interest.

## Supporting information


Appendix S1

Figure S1

Table S1

Table S2

Table S3

Table S4

Table S5

Table S6

Table S7

## Data Availability

The data that support the findings of this study are openly available in TCGA and GEO databases.
