# Supplementary material for: Downregulated ferroptosis‐related gene SQLE facilitates temozolomide chemoresistance, and invasion and affects immune regulation in glioblastoma
Source: CNS Neurosci Ther. 2022 Aug 13;28(12):2104–15. doi: 10.1111/cns.13945 (PMC9627366; doi:10.1111/cns.13945)
Supplement: Supplementary file 1 — Appendix S1 [file CNS-28-2104-s006.pdf]

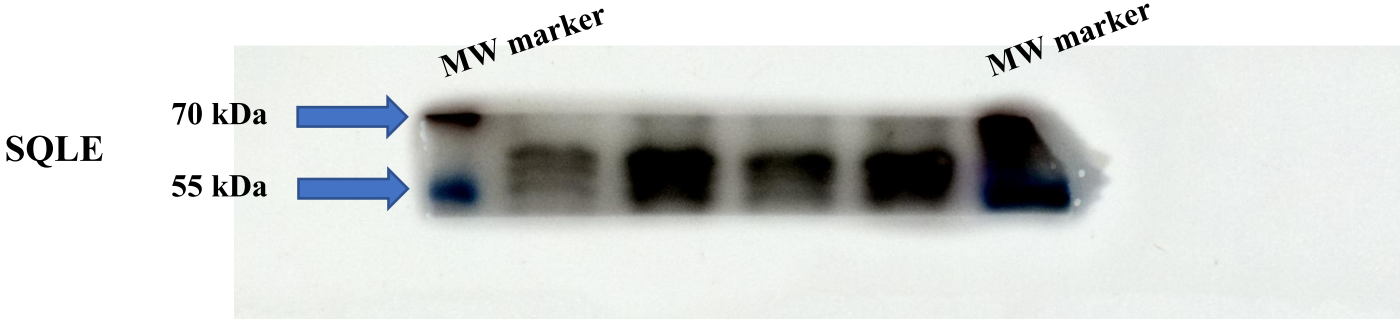

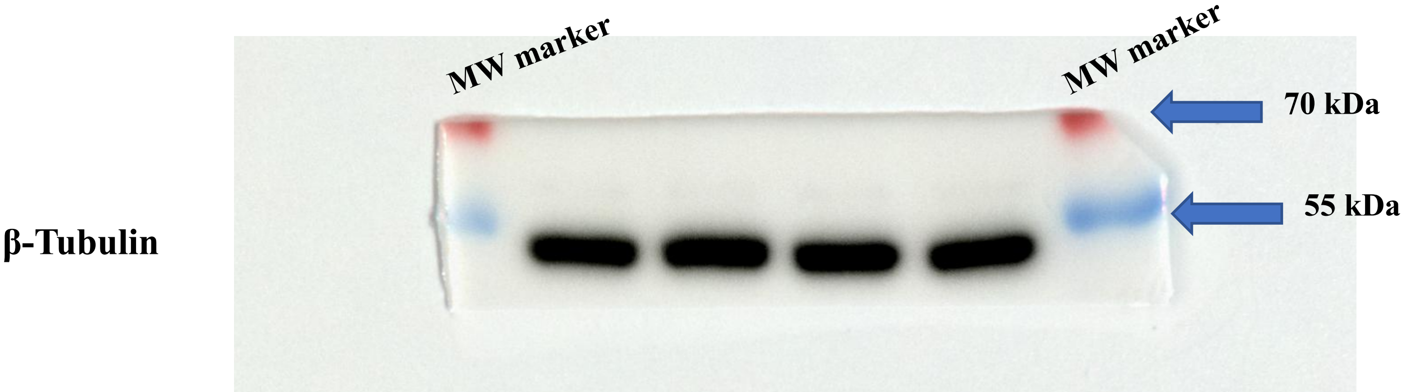

Full unedited gel/blot for Figure 4A in the manuscript

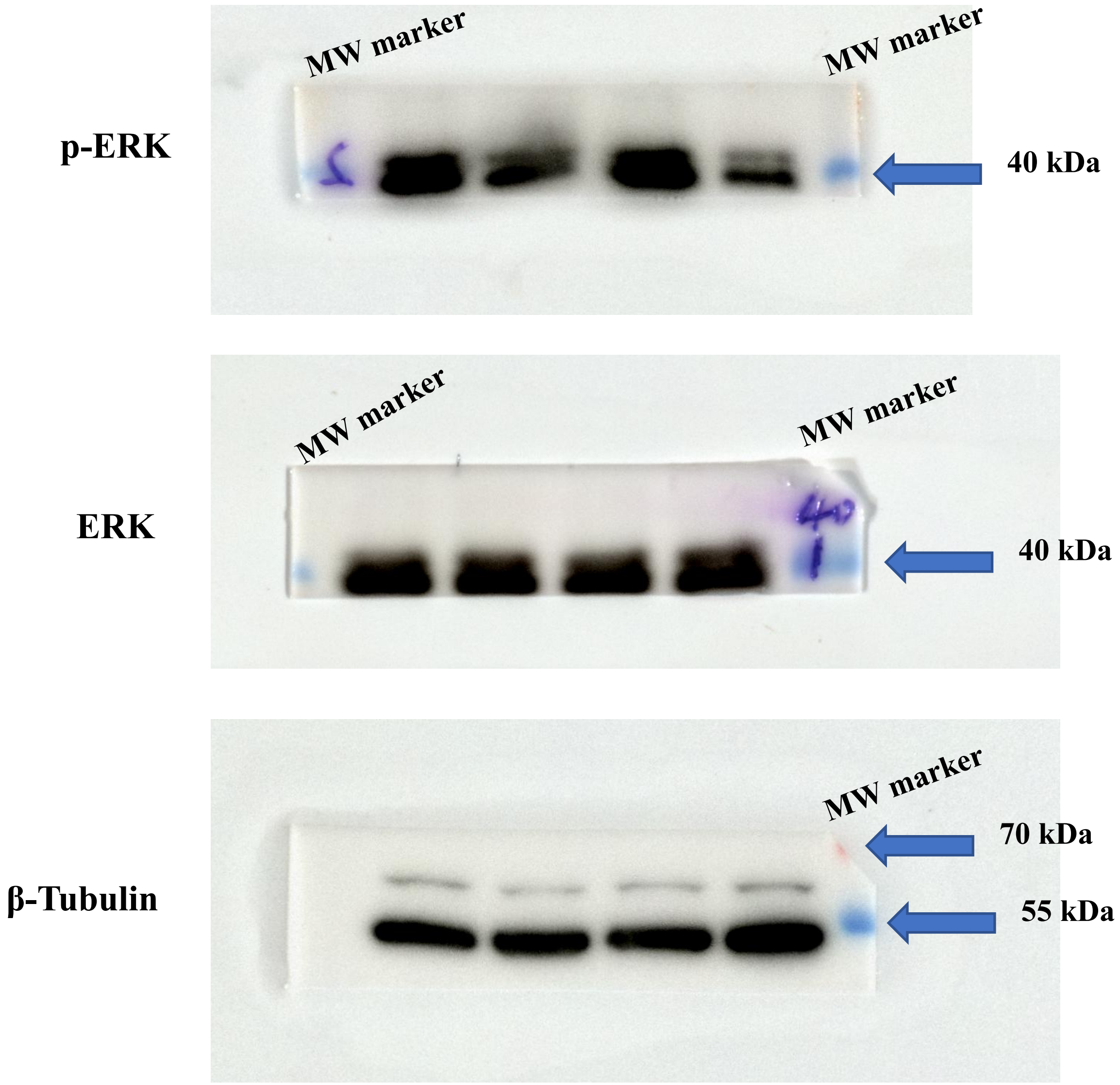

Full unedited gel/blot for Figure 4B in the manuscript

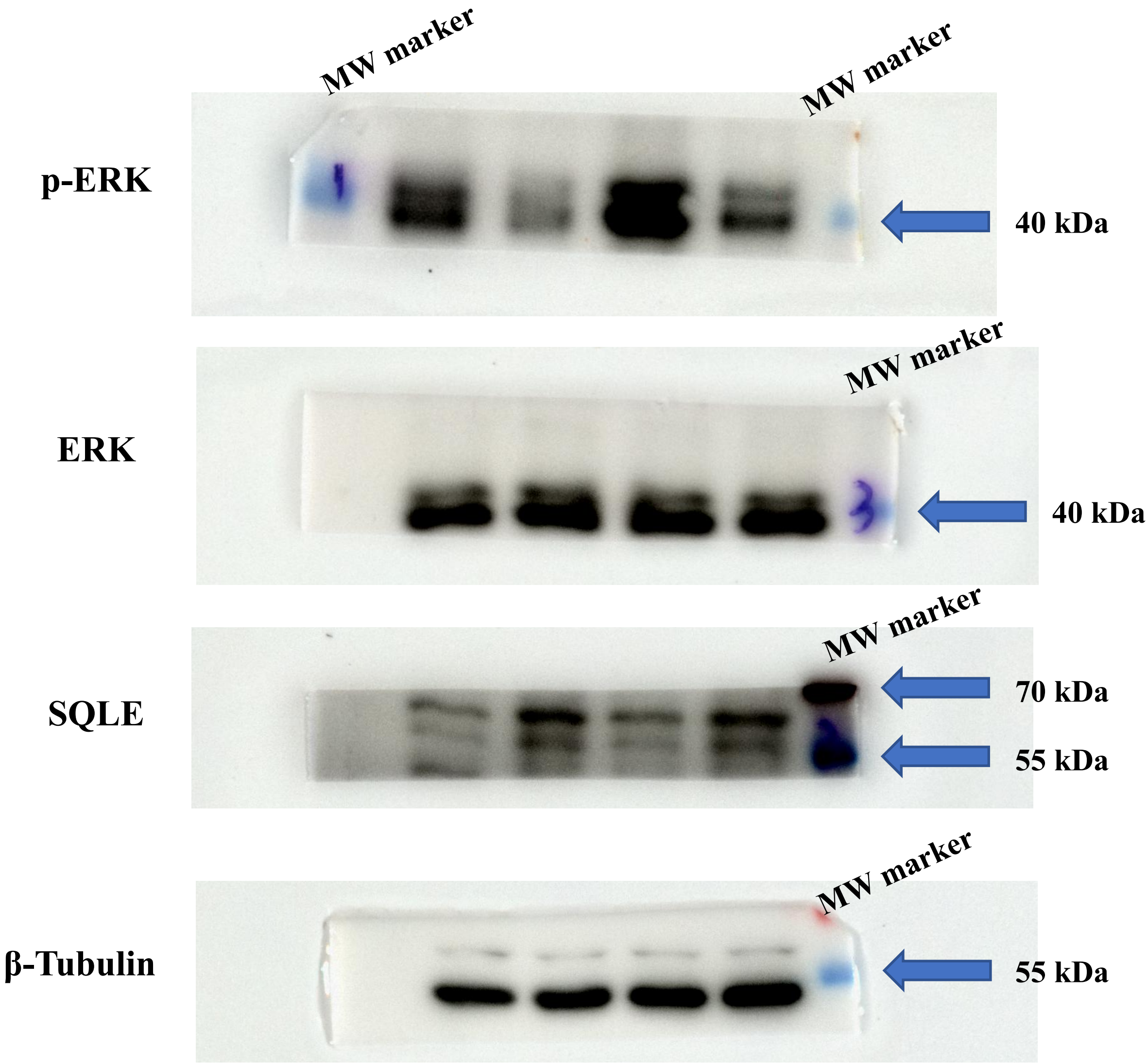

Full unedited gel/blot for Figure 4C in the manuscript

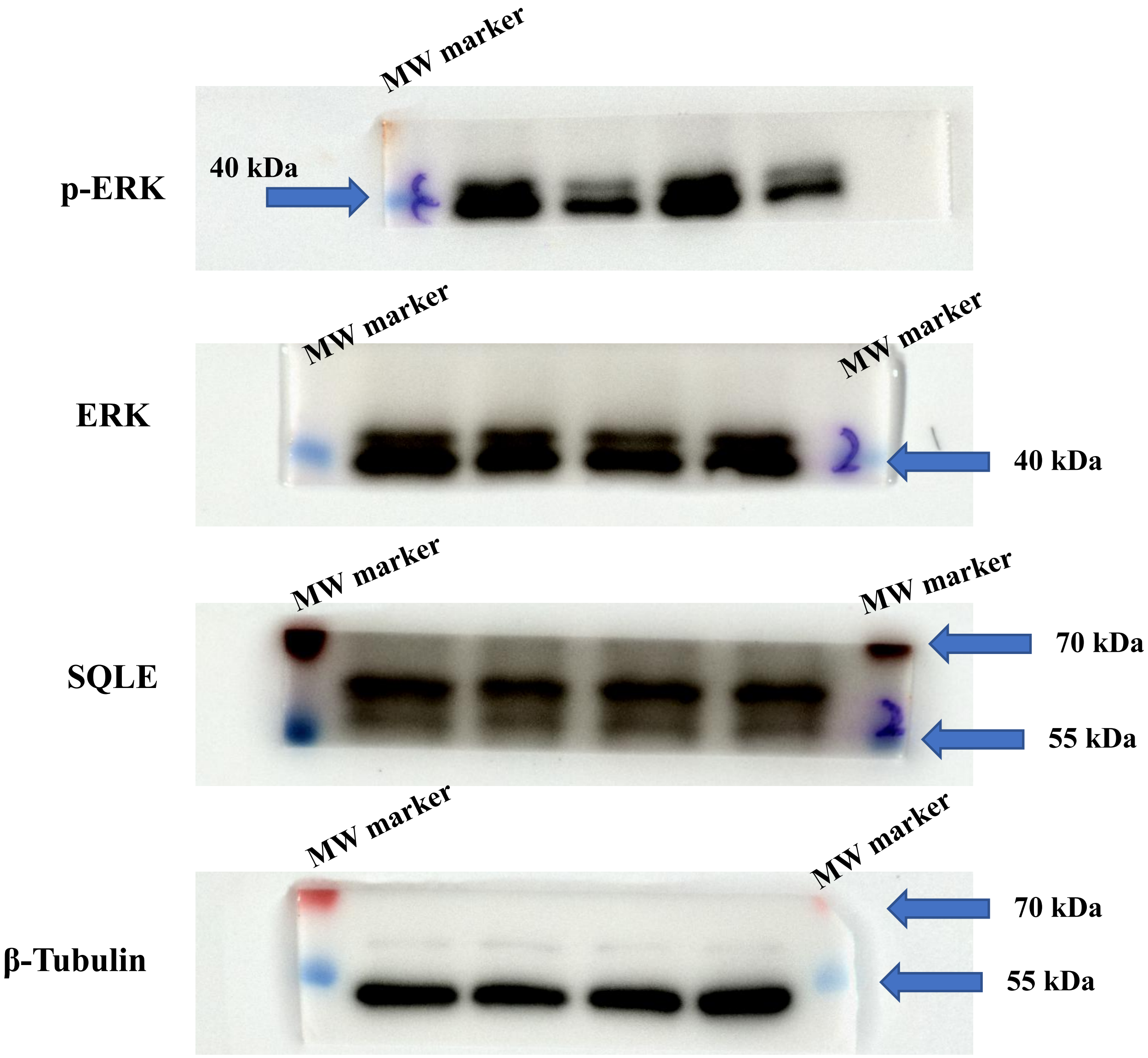

Full unedited gel/blot for Figure 4D in the manuscript

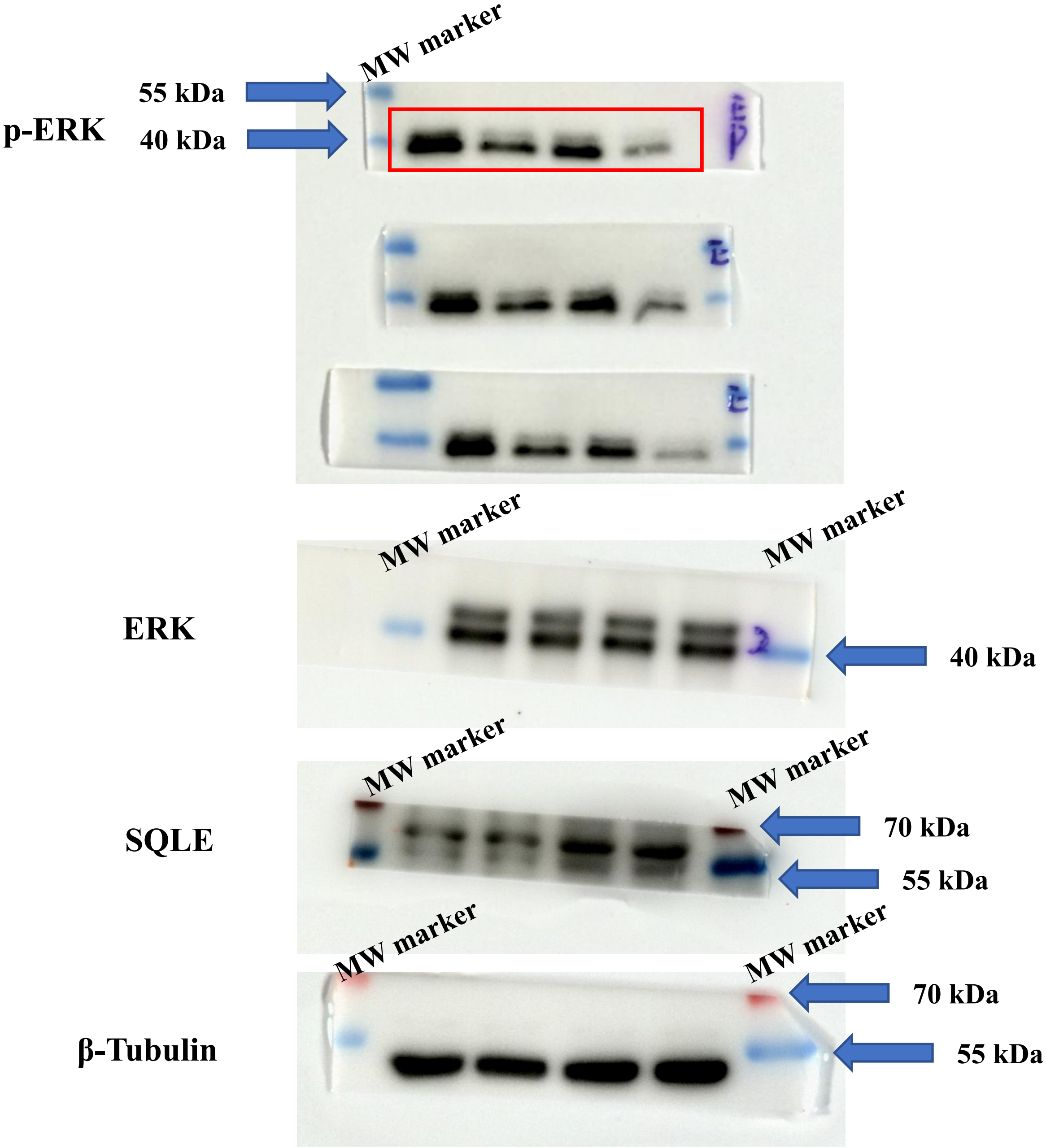

Full unedited gel/blot for Figure 4E in the manuscript

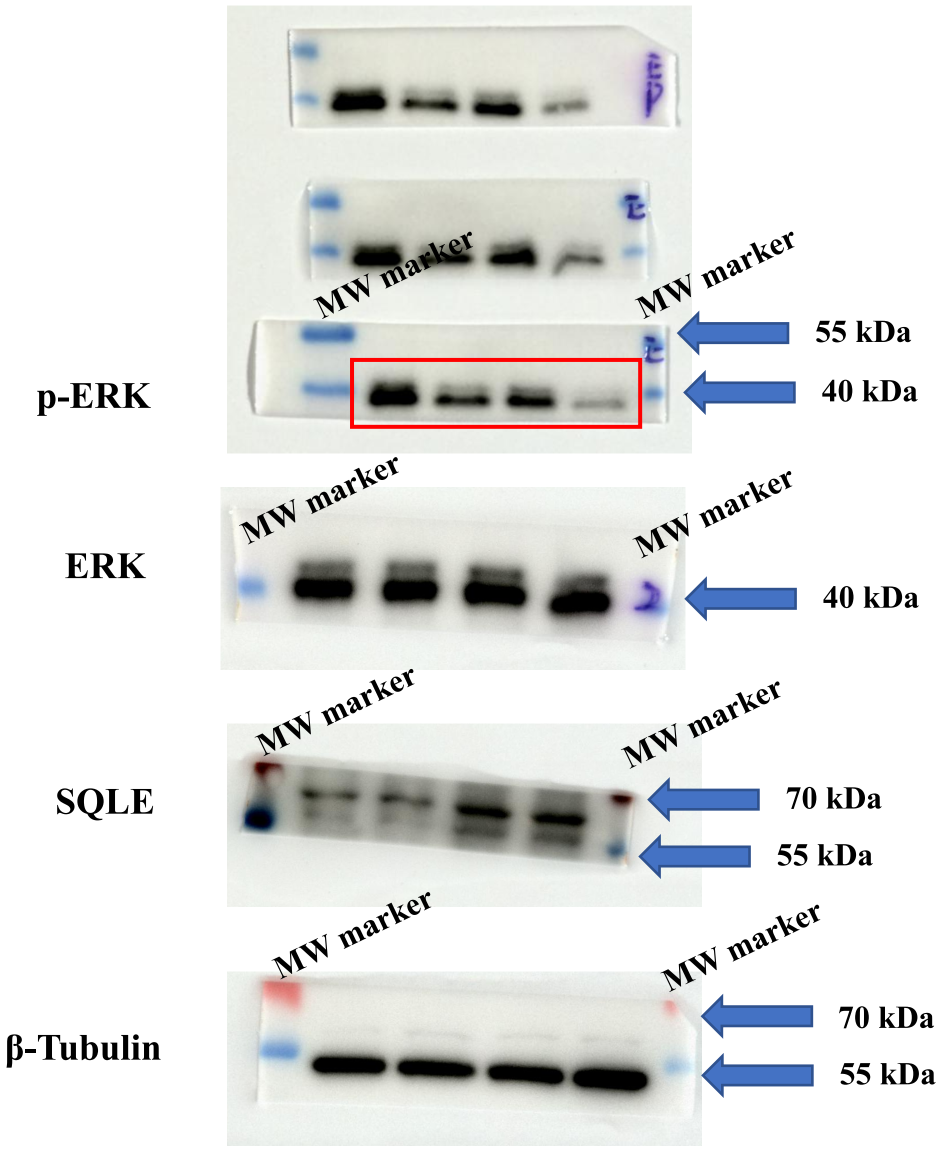

Full unedited gel/blot for Figure 4F in the manuscript

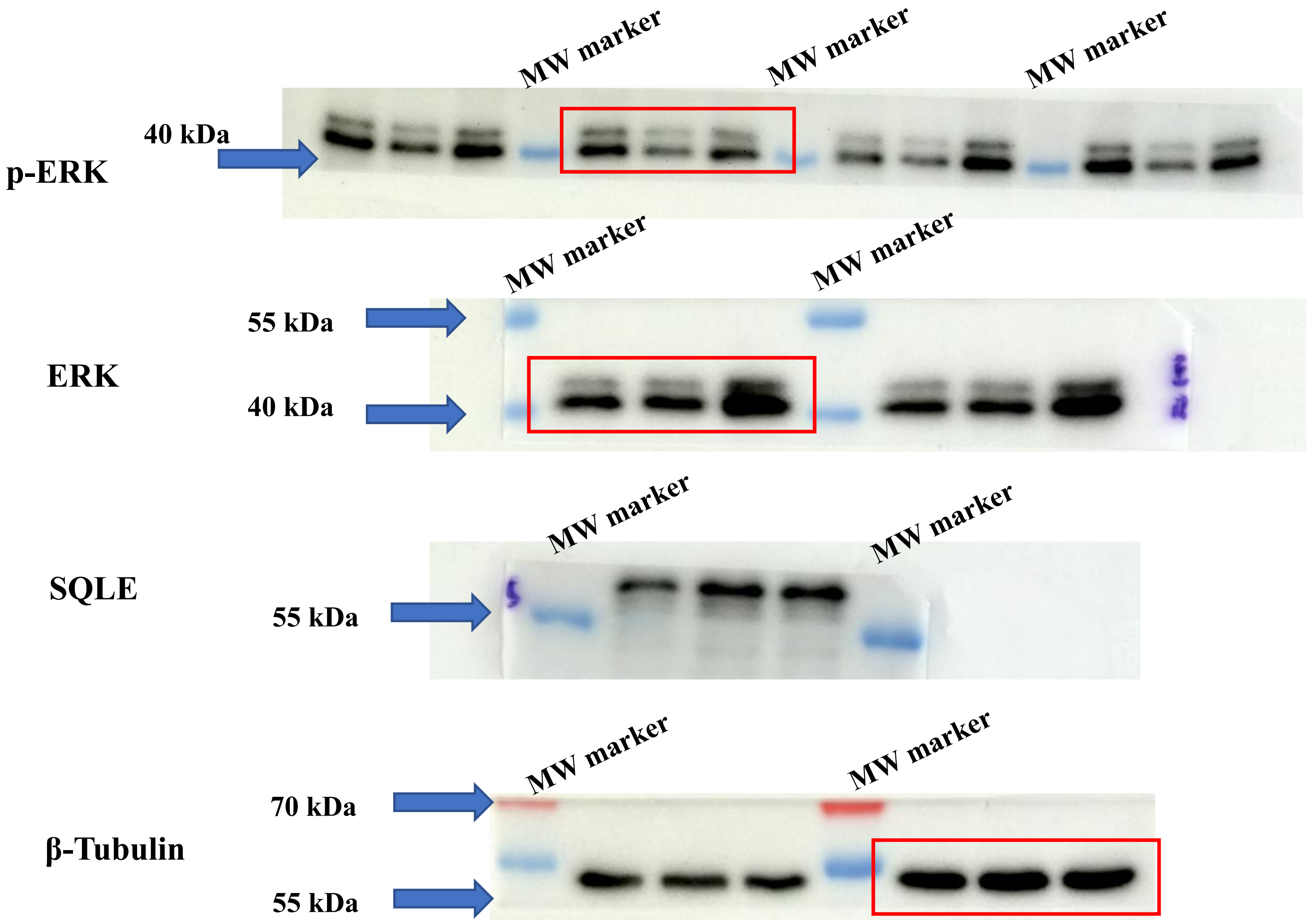

Full unedited gel/blot for Supplementary Figure S2A in the manuscript

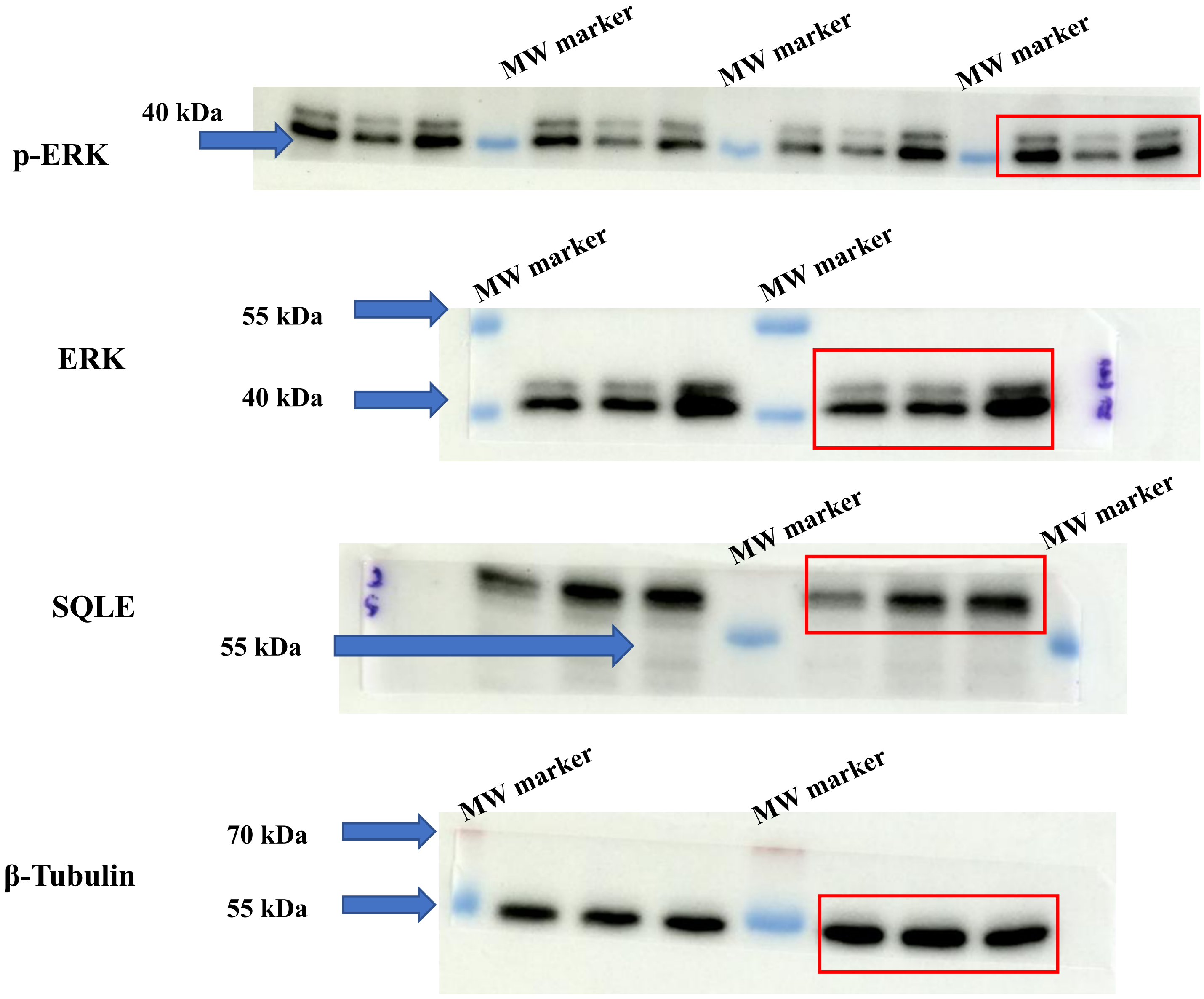

Full unedited gel/blot for Supplementary Figure S2B in the manuscript
