# Supplementary material for: Downregulated ferroptosis‐related gene SQLE facilitates temozolomide chemoresistance, and invasion and affects immune regulation in glioblastoma
Source: CNS Neurosci Ther. 2022 Aug 13;28(12):2104–15. doi: 10.1111/cns.13945 (PMC9627366; doi:10.1111/cns.13945)
Supplement: Supplementary file 2 — Figure S1 [file CNS-28-2104-s009.docx]

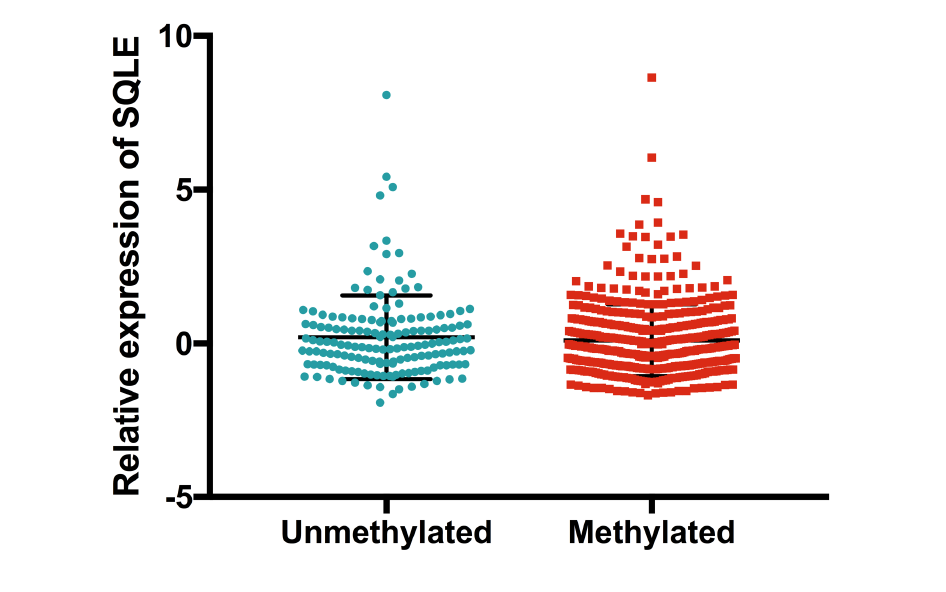


**Supplementary Figure S1.** The expression of SQLE between MGMT promoter unmethylated group and methylated group in glioma (cBioportal).


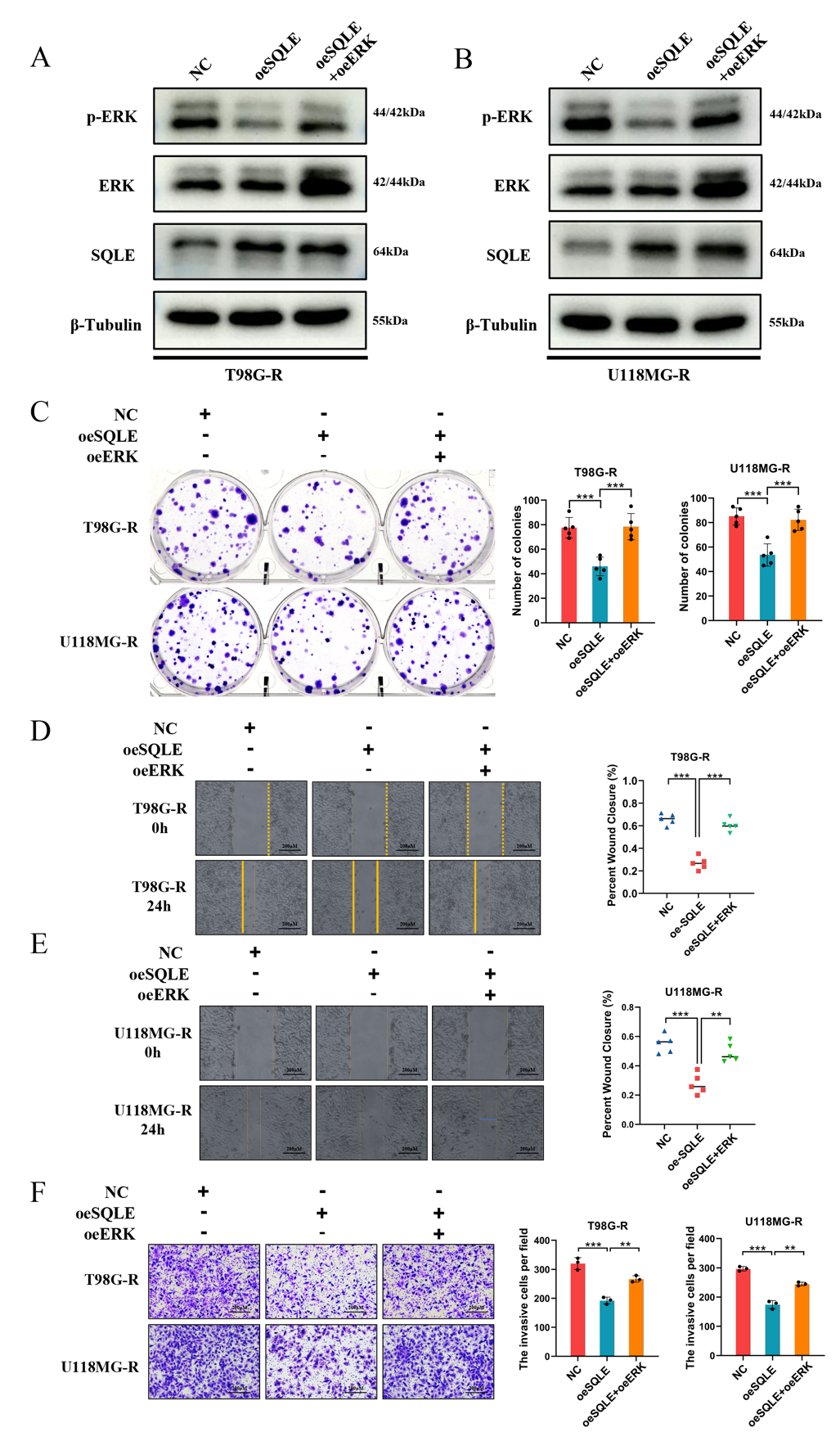


**Supplementary Figure S2.** SQLE inhibits the metastatic ability and proliferation of GBM cells via ERK pathway. (A-B) Western Blot for SQLE, p-ERK, and ERK in T98G-R and U118MG-R cells transfected with NC, SQLE plasmid, and SQLE plasmid plus ERK plasmid. Colony formation assay (C), wound healing assay (D-E), and transwell invasion assay (F) for T98G-R and U118MG-R cells treated with NC, SQLE plasmid, and SQLE plasmid plus ERK plasmid.


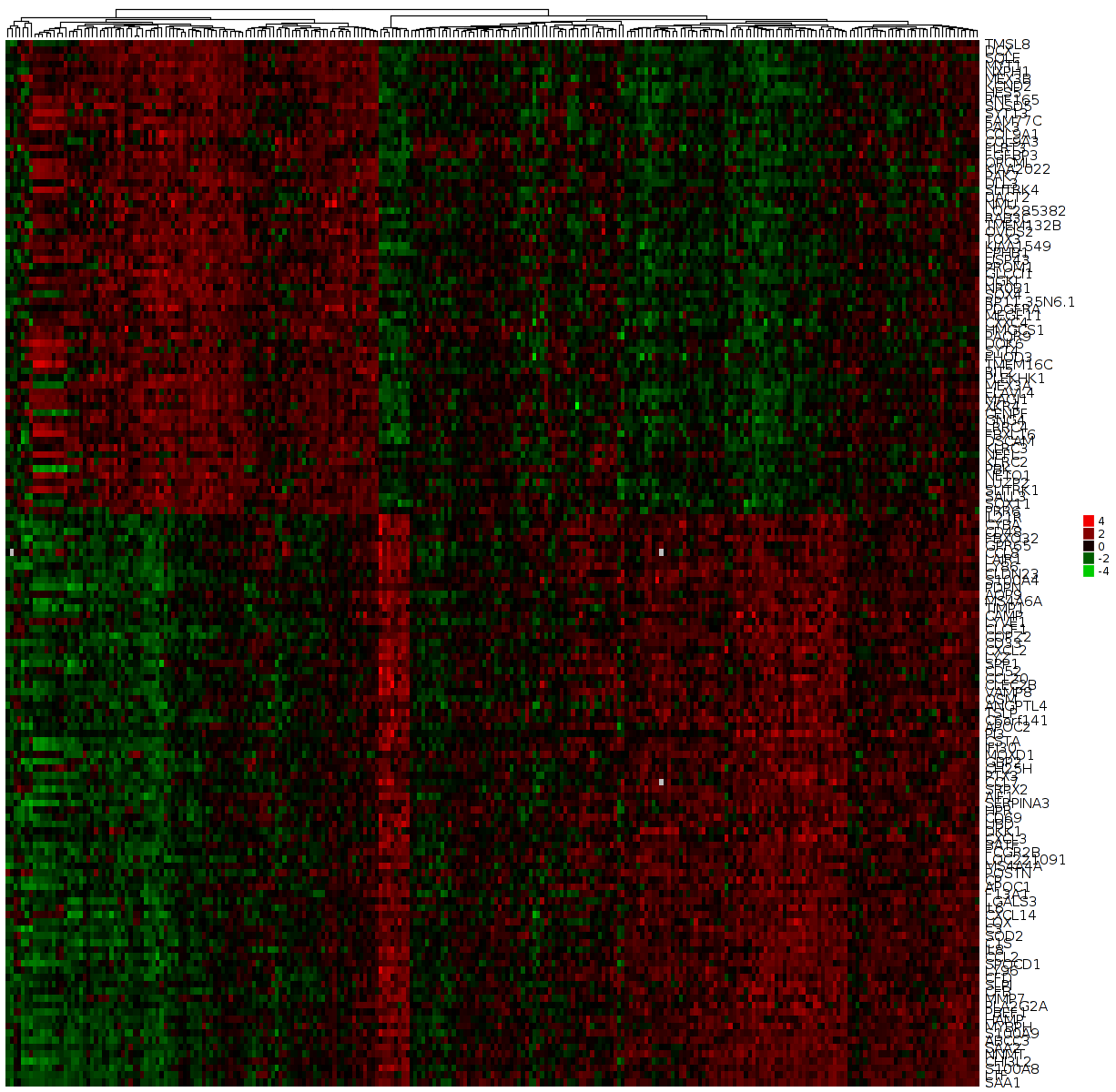


**Supplementary Figure S3.** The heatmap showed the co-expression genes of SQLE via GlioVis.


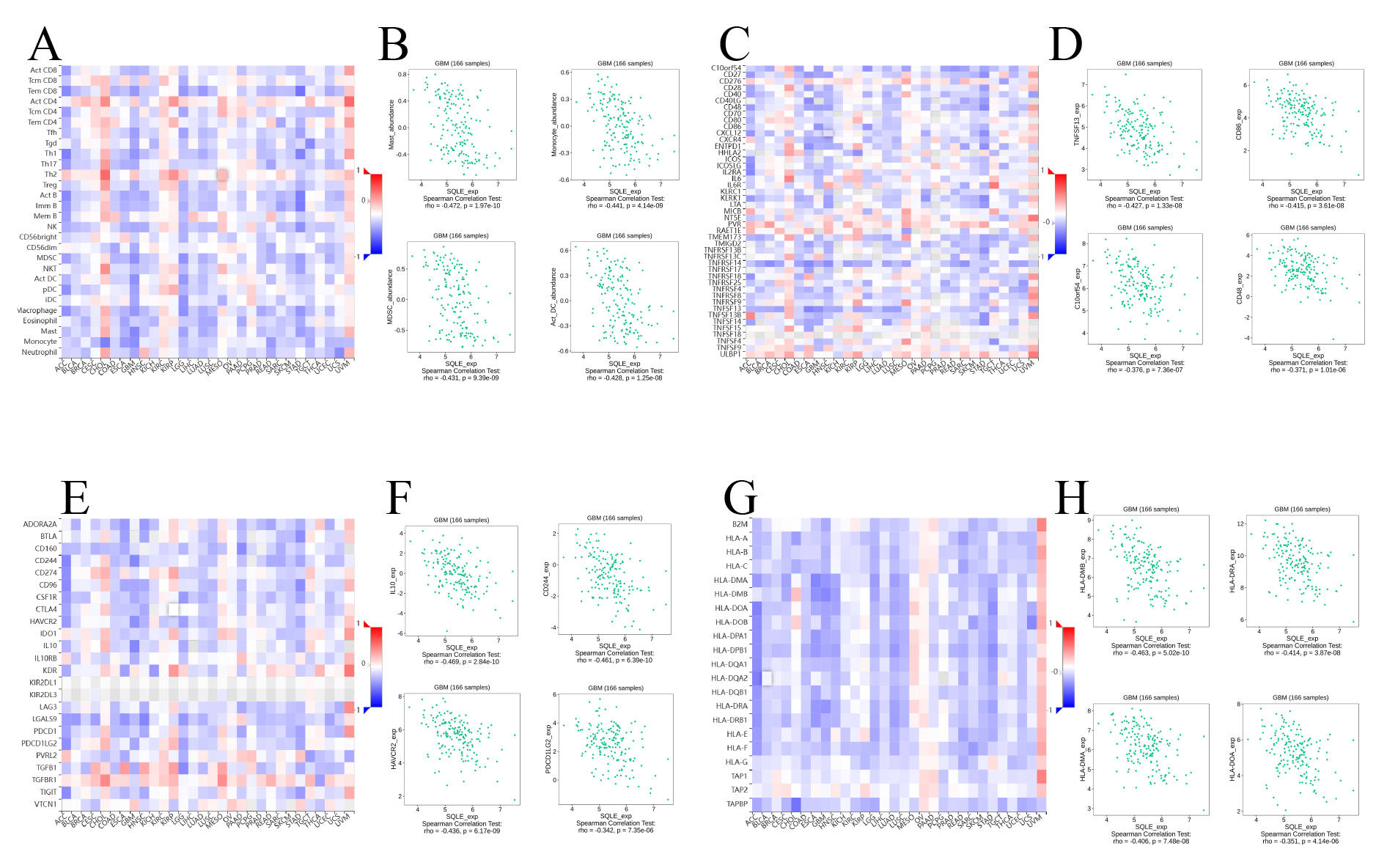


**Supplementary Figure S4.** Correlation analysis between SQLE expression and immune molecules in GBM. (A-B) The association between the expression of SQLE and TILs, and the top 4-ranked TILs molecules exhibiting more relevance with SQLE. (C-D) The association between the expression of SQLE and immunostimulators, and the top 4-ranked immunostimulators molecules exhibiting more relevance with SQLE. (E-F) The association between the expression of SQLE and immunoinhibitors, and the top 4-ranked immunoinhibitors molecules exhibiting more relevance with SQLE. (G-H) The association between the expression of SQLE and MHCs, and the top 4-ranked MHCs molecules exhibiting more relevance with SQLE.
