# Supplementary material for: Downregulated ferroptosis‐related gene SQLE facilitates temozolomide chemoresistance, and invasion and affects immune regulation in glioblastoma
Source: CNS Neurosci Ther. 2022 Aug 13;28(12):2104–15. doi: 10.1111/cns.13945 (PMC9627366; doi:10.1111/cns.13945)
Supplement: Supplementary file 3 — Table S1 [file CNS-28-2104-s001.docx]

**Supplementary Table S1.** The main bioinformatics tools used to analyze the role of SQLE in GBM.

| **Database** | **Samples** | **URL** | **References** |
| --- | --- | --- | --- |
| FunRich | - | http://www.funrich.org | [33] |
| GlioVis | Tissues | http://gliovis.bioinfo.cnio.es/ | [34] |
| THPA | Tissues | http://www.proteinatlas.org/ | [35] |
| CCLE | Cell lines | https://portals.broadinstitute.org/ccle/ | [36] |
| OncoScape | Cell lines | https://oncoscape.nki.nl/ | [37] |
| CellMinerCDB | Cell lines | https://discover.nci.nih.gov/rsconnect/cellminercdb/ | [38] |
| STRING | - | http://string-db.org/ | [39] |
| GSEA | - | https://www.gsea-msigdb.org/gsea/index.jsp | [41] |
| TISIDB | Tissues | http://cis.hku.hk/TISIDB | [42] |
