# Supplementary material for: Downregulated ferroptosis‐related gene SQLE facilitates temozolomide chemoresistance, and invasion and affects immune regulation in glioblastoma
Source: CNS Neurosci Ther. 2022 Aug 13;28(12):2104–15. doi: 10.1111/cns.13945 (PMC9627366; doi:10.1111/cns.13945)
Supplement: Supplementary file 4 — Table S2 [file CNS-28-2104-s002.docx]

**Supplementary Table S2.** The main characteristics of 3 gene expression profiles by microarray.

| **GEO^†^**  **datasets** | **Platform** | **Samples size** | | **DEGs^#^** | **Co-DEGs** | **Submission date** | **References** |
| --- | --- | --- | --- | --- | --- | --- | --- |
|  |  | **Control** | **Treatment** |  |  |  |  |
| GSE47809 | GPL570 | 3 | 4 | 5475 | 156 | Jun 10, 2013 | N/A^∆^ |
| GSE80729 | GPL10558 | 3 | 3 | 1875 |  | Apr 27, 2016 |  |
| GSE65363 | GPL570 | 3 | 3 | 3961 |  | Jan 28, 2015 | N/A^∆^ |

Note:

**†** GEO: Gene Expression Omnibus datasets.

# DEGs: differentially expressed genes.

∆ Further information can be found at https://www.ncbi.nlm.nih.gov/geo/.
