# Supplementary material for: Downregulated ferroptosis‐related gene SQLE facilitates temozolomide chemoresistance, and invasion and affects immune regulation in glioblastoma
Source: CNS Neurosci Ther. 2022 Aug 13;28(12):2104–15. doi: 10.1111/cns.13945 (PMC9627366; doi:10.1111/cns.13945)
Supplement: Supplementary file 5 — Table S3 [file CNS-28-2104-s005.docx]

**Supplementary Table S3.** The ferroptosis related genes.

| SQLE | FANCD2 | HMGCR | MT1G | CD44 | AKR1C3 | GPX4 | STEAP3 | HSPB1 | CRYAB |
| --- | --- | --- | --- | --- | --- | --- | --- | --- | --- |
| GCLM | FTH1 | TP53 | NOX1 | HMOX1 | G6PD | ALOX12 | ZEB1 | SLC7A11 | TFRC |
| AKR1C1 | GOT1 | KEAP1 | ALOX5 | EMC2 | CS | PGD | PHKG2 | PEBP1 | ACSL3 |
| NCOA4 | ALOX15 | ACACA | CISD1 | HSBP1 | SLC1A5 | AKR1C2 | GSS | GCLC | RPL8 |
| FDFT1 | FADS2 | PTGS2 | ACSL4 | AIFM2 | CARS | GLS2 | CBS | SAT1 | ABCC1 |
| CHAC1 | NQO1 | DPP4 | IREB2 | NFS1 | LPCAT3 | ACO1 | NFE2L2 | ATP5MC3 | ACSF2 |
