# Supplementary material for: Downregulated ferroptosis‐related gene SQLE facilitates temozolomide chemoresistance, and invasion and affects immune regulation in glioblastoma
Source: CNS Neurosci Ther. 2022 Aug 13;28(12):2104–15. doi: 10.1111/cns.13945 (PMC9627366; doi:10.1111/cns.13945)
Supplement: Supplementary file 6 — Table S4 [file CNS-28-2104-s007.docx]

**Supplementary Table S4.** The 156 co-differentially expressed genes (co-DEGs) in all three GEO datasets (GSE47809, GSE65363 and GSE80729).

| SQLE | FANCD2 | SMS | ASPH | DNM1L | MELTF | HAUS2 | SLC38A10 | CDK1 | ARHGAP28 |
| --- | --- | --- | --- | --- | --- | --- | --- | --- | --- |
| OSMR | N4BP2L2 | CMBL | TUBD1 | SSX3 | BTG2 | ABCA1 | AGAP1 | FAM111A | TMEM106C |
| TP53INP1 | YME1L1 | EXD2 | C10orf88 | BNIP3 | SALL1 | TNS1 | PPM1A | CKAP2 | CASP7 |
| G3BP1 | CCDC15 | YPEL1 | CCNG1 | KMT2E | TMEM43 | CHRNA10 | CX3CR1 | GPSM2 | PPHLN1 |
| VEGFA | PARVB | SYNCRIP | TPR | ZNF337 | SPTBN1 | SLC5A3 | NFIC | FST | PDE8A |
| IPO7 | ING3 | BRD2 | GRM2 | HELLS | BOLA2 | SOCS3 | EFCAB7 | PATZ1 | NRG1 |
| BTG4 | GAB1 | LCE1E | MSI2 | TMCC1 | BTBD7 | RCAN1 | C16orf72 | EPB41L5 | VANGL1 |
| GPER1 | ZNF493 | POLQ | SIPA1L2 | MYCN | SNORD89 | ATP6V0E1 | FOXN3 | OSBPL3 | TIGAR |
| BROX | WHRN | BAX | AQP4 | ZNF491 | DTNA | USP12 | SFRP1 | AKT3 | ARPP21 |
| JAG1 | HNF4A | ABCG1 | FNIP2 | MCL1 | CDK5R1 | G3BP2 | WDR27 | ARL6IP6 | HNRNPA2B1 |
| NIM1K | SDCCAG3 | PLN | ELMSAN1 | STAT1 | LOC283788 | MYL10 | NAPG | PDE4A | STIM2 |
| PPM1D | PPFIBP1 | ZMAT3 | LOC81691 | CYP1B1 | IPPK | THSD7A | PDLIM5 | CENPF | HDAC9 |
| ELK4 | STXBP6 | ZNF148 | WTAP | PPIP5K1 | HNRNPC | MAGEE1 | PRDM1 | CLIP1 | RPS15A |
| GINS4 | CXorf57 | EVI2A | ASH1L | FSCN1 | CYP2E1 | ZADH2 | KIF13A | ALS2 | SORBS2 |
| ATF5 | KCTD1 | SLC34A1 | HECTD2 | PCDHB6 | RTTN | PGK1 | LARP4 | DHX9 | RFXAP |
| ATM | PARP11 | ESR1 | THRA | KLF3 | CEP295 |  |  |  |  |
