# Supplementary material for: Downregulated ferroptosis‐related gene SQLE facilitates temozolomide chemoresistance, and invasion and affects immune regulation in glioblastoma
Source: CNS Neurosci Ther. 2022 Aug 13;28(12):2104–15. doi: 10.1111/cns.13945 (PMC9627366; doi:10.1111/cns.13945)
Supplement: Supplementary file 7 — Table S5 [file CNS-28-2104-s004.docx]

**Supplementary Table S5.** Correlation of SQLE with clinicopathological parameters in glioma.

| **Characteristics** | **Total(N)** | **Odds Ratio(OR)** | **P value** |
| --- | --- | --- | --- |
| Age (>60 vs. <=60) | 696 | 0.916 (0.633-1.323) | 0.639 |
| Gender (Male vs. Female) | 696 | 0.829 (0.613-1.119) | 0.221 |
| WHO grade (G4 vs. G2&G3) | 635 | 0.565 (0.394-0.806) | 0.002* |
| IDH status (Mut vs. WT) | 686 | 1.325 (0.969-1.813) | 0.078 |
| 1p/19q codeletion (non-codel vs. codel) | 689 | 0.226 (0.151-0.331) | <0.001* |
