# Supplementary material for: Downregulated ferroptosis‐related gene SQLE facilitates temozolomide chemoresistance, and invasion and affects immune regulation in glioblastoma
Source: CNS Neurosci Ther. 2022 Aug 13;28(12):2104–15. doi: 10.1111/cns.13945 (PMC9627366; doi:10.1111/cns.13945)
Supplement: Supplementary file 8 — Table S6 [file CNS-28-2104-s003.docx]

**Supplementary Table S6.** The correlation of CDKN1A expression and TMZ activity in glioma cell lines.

| Cell Line | expSQLE_gdscDec15 | actTemozolomide_gdscDec15 | tissues | OncoTree1 | OncoTree2 | OncoTree3 | OncoTree4 | EMT |
| --- | --- | --- | --- | --- | --- | --- | --- | --- |
| D-423MG | 6.942734827 | 4.457122882 | GBM | Brain_CNS | NA | NA | NA | Mesenchymal |
| CHP-126 | 8.979781067 | 4.411791913 | NA | Brain_CNS | Embryonal Tumor (EMBT) | Neuroblastoma (NBL) | NA | Epithelial-Mesenchymal |
| GI-1 | 7.731438291 | 4.36940646 | OTHER | Brain_CNS | Diffuse Glioma (DIFG) | Glioblastoma (GB) | Gliosarcoma (GSARC) | Mesenchymal |
| GI-ME-N | 7.562002961 | 4.320052233 | NA | Brain_CNS | Embryonal Tumor (EMBT) | Neuroblastoma (NBL) | NA | Mesenchymal |
| 8-MG-BA | 8.159152749 | 4.306662453 | GBM | Brain_CNS | Diffuse Glioma (DIFG) | Astrocytoma (ASTR) | NA | Mesenchymal |
| MOG-G-UVW | 7.120650444 | 4.223093847 | OTHER | Brain_CNS | Diffuse Glioma (DIFG) | Astrocytoma (ASTR) | NA | Mesenchymal |
| U251 | 6.16474571 | 4.10723012 | GBM | Brain_CNS | Diffuse Glioma (DIFG) | Astrocytoma (ASTR) | NA | Mesenchymal |
| LNZTA3WT4 | 8.764969328 | 4.069687611 | GBM | Brain_CNS | NA | NA | NA | Mesenchymal |
| SF539 | 9.58686708 | 4.02633342 | OTHER | Brain_CNS | NA | NA | NA | Mesenchymal |
| CHP-212 | 8.525586079 | 4.001707117 | NA | Brain_CNS | Embryonal Tumor (EMBT) | Neuroblastoma (NBL) | NA | Mesenchymal |
| D-566MG | 7.694851017 | 3.971000585 | GBM | Brain_CNS | NA | NA | NA | Mesenchymal |
| IMR-5 | 9.195637225 | 3.970117652 | NA | Brain_CNS | Embryonal Tumor (EMBT) | Neuroblastoma (NBL) | NA | Mesenchymal |
| LN-229 | 6.739703374 | 3.909891052 | GBM | Brain_CNS | Diffuse Glioma (DIFG) | Astrocytoma (ASTR) | NA | Mesenchymal |
| M059J | 7.831485363 | 3.909388216 | GBM | Brain_CNS | NA | NA | NA | Mesenchymal |
| 42-MG-BA | 9.505648745 | 3.869928354 | GBM | Brain_CNS | Diffuse Glioma (DIFG) | Astrocytoma (ASTR) | NA | Mesenchymal |
| A172 | 7.59437062 | 3.852948153 | GBM | Brain_CNS | Diffuse Glioma (DIFG) | Astrocytoma (ASTR) | NA | Mesenchymal |
| NB10 | 7.325302994 | 3.844685649 | NA | Brain_CNS | Embryonal Tumor (EMBT) | Neuroblastoma (NBL) | NA | Mesenchymal |
| KS-1 | 7.657774948 | 3.835992551 | GBM | Brain_CNS | Diffuse Glioma (DIFG) | Astrocytoma (ASTR) | NA | Mesenchymal |
| TGW | 8.913860811 | 3.805670372 | NA | Brain_CNS | Embryonal Tumor (EMBT) | Neuroblastoma (NBL) | NA | Mesenchymal |
| SF268 | 8.337351707 | 3.79709332 | GBM | Brain_CNS | NA | NA | NA | Mesenchymal |
| NB12 | 8.526946044 | 3.780198502 | NA | Brain_CNS | Embryonal Tumor (EMBT) | Neuroblastoma (NBL) | NA | Mesenchymal |
| GB-1 | 5.621625774 | 3.772711272 | GBM | Brain_CNS | Diffuse Glioma (DIFG) | Astrocytoma (ASTR) | NA | Mesenchymal |
| ACN | 7.374671393 | 3.764559619 | NA | Brain_CNS | Embryonal Tumor (EMBT) | Neuroblastoma (NBL) | NA | Mesenchymal |
| SW1088 | 8.524860897 | 3.747114609 | GBM | Brain_CNS | NA | NA | NA | Mesenchymal |
| Daoy | 7.58773415 | 3.720243001 | OTHER | Brain_CNS | Embryonal Tumor (EMBT) | Medulloblastoma (MBL) | NA | Mesenchymal |
| SF295 | 6.972695749 | 3.718894673 | GBM | Brain_CNS | Diffuse Glioma (DIFG) | Astrocytoma (ASTR) | NA | Mesenchymal |
| Becker | 6.250622048 | 3.687336594 | GBM | Brain_CNS | Diffuse Glioma (DIFG) | Astrocytoma (ASTR) | NA | Mesenchymal |
| NBsusSR | 8.166359708 | 3.67770147 | NA | Brain_CNS | Embryonal Tumor (EMBT) | Neuroblastoma (NBL) | NA | Mesenchymal |
| T98G | 8.231040251 | 3.673743654 | GBM | Brain_CNS | Diffuse Glioma (DIFG) | Astrocytoma (ASTR) | NA | Mesenchymal |
| SK-N-FI | 7.777774007 | 3.655012411 | NA | Brain_CNS | Embryonal Tumor (EMBT) | Neuroblastoma (NBL) | NA | Mesenchymal |
| H4 | 6.362291706 | 3.642173587 | CANNOT_CLASSIFY | Brain_CNS | NA | NA | NA | Mesenchymal |
| LN-18 | 7.569954325 | 3.627903256 | GBM | Brain_CNS | Diffuse Glioma (DIFG) | Astrocytoma (ASTR) | NA | Mesenchymal |
| SW1783 | 8.627548577 | 3.580160337 | OTHER | Brain_CNS | Diffuse Glioma (DIFG) | Astrocytoma (ASTR) | NA | Mesenchymal |
| AM-38 | 8.09705021 | 3.571708523 | GBM | Brain_CNS | Diffuse Glioma (DIFG) | Astrocytoma (ASTR) | NA | Mesenchymal |
| SK-MG-1 | 7.03798612 | 3.571487211 | GBM | Brain_CNS | NA | NA | NA | Mesenchymal |
| KNS-42 | 6.446677268 | 3.569004088 | CANNOT_CLASSIFY | Brain_CNS | NA | NA | NA | Mesenchymal |
| NB7 | 8.14570394 | 3.559813837 | NA | Brain_CNS | Embryonal Tumor (EMBT) | Neuroblastoma (NBL) | NA | Epithelial-Mesenchymal |
| SK-N-SH | 6.586601406 | 3.555003162 | OTHER | Brain_CNS | Embryonal Tumor (EMBT) | Neuroblastoma (NBL) | NA | Mesenchymal |
| NB13 | 9.830751999 | 3.554254371 | NA | Brain_CNS | Embryonal Tumor (EMBT) | Neuroblastoma (NBL) | NA | Mesenchymal |
| SF126 | 7.440160054 | 3.552048893 | GBM | Brain_CNS | Diffuse Glioma (DIFG) | Astrocytoma (ASTR) | NA | Mesenchymal |
| KELLY | 8.516772216 | 3.551092988 | OTHER | Brain_CNS | Embryonal Tumor (EMBT) | Neuroblastoma (NBL) | NA | Mesenchymal |
| NB14 | 9.586691975 | 3.533843169 | NA | Brain_CNS | Embryonal Tumor (EMBT) | Neuroblastoma (NBL) | NA | Mesenchymal |
| D-283MED | 8.88711729 | 3.522341368 | OTHER | Brain_CNS | Embryonal Tumor (EMBT) | Medulloblastoma (MBL) | NA | Mesenchymal |
| GAMG | 7.890189109 | 3.503993409 | CANNOT_CLASSIFY | Brain_CNS | NA | NA | NA | Mesenchymal |
| NMC-G1 | 6.191884163 | 3.482792441 | CANNOT_CLASSIFY | Brain_CNS | NA | NA | NA | Mesenchymal |
| NB5 | 8.774857903 | 3.46496566 | NA | Brain_CNS | Embryonal Tumor (EMBT) | Neuroblastoma (NBL) | NA | Mesenchymal |
| SK-N-DZ | 8.486637146 | 3.444970413 | NA | Brain_CNS | Embryonal Tumor (EMBT) | Neuroblastoma (NBL) | NA | Mesenchymal |
| Hs-683 | 7.1751097 | 3.444192998 | CANNOT_CLASSIFY | Brain_CNS | NA | NA | NA | Mesenchymal |
| ONS-76 | 6.487235765 | 3.443439255 | OTHER | Brain_CNS | Embryonal Tumor (EMBT) | Medulloblastoma (MBL) | NA | Mesenchymal |
| CCF-STTG1 | 7.275694997 | 3.435206459 | NA | Brain_CNS | Diffuse Glioma (DIFG) | Astrocytoma (ASTR) | NA | Mesenchymal |
| no-10 | 7.63677681 | 3.41844892 | OTHER | Brain_CNS | NA | NA | NA | Mesenchymal |
| YH-13 | 7.205714232 | 3.416985966 | GBM | Brain_CNS | Diffuse Glioma (DIFG) | Astrocytoma (ASTR) | NA | Mesenchymal |
| YKG-1 | 7.541353305 | 3.416737384 | GBM | Brain_CNS | Diffuse Glioma (DIFG) | Astrocytoma (ASTR) | NA | Mesenchymal |
| D-263MG | 8.163360071 | 3.412437204 | GBM | Brain_CNS | NA | NA | NA | Mesenchymal |
| KALS-1 | 7.75191236 | 3.402783844 | CANNOT_CLASSIFY | Brain_CNS | NA | NA | NA | Mesenchymal |
| GOTO | 8.589630832 | 3.390466237 | NA | Brain_CNS | Embryonal Tumor (EMBT) | Neuroblastoma (NBL) | NA | Mesenchymal |
| NB1 | 8.948971711 | 3.384198185 | NA | Brain_CNS | Embryonal Tumor (EMBT) | Neuroblastoma (NBL) | NA | Mesenchymal |
| KINGS-1 | 6.8709257 | 3.355789886 | OTHER | Brain_CNS | NA | NA | NA | Mesenchymal |
| MOG-G-CCM | 7.641892798 | 3.344664903 | OTHER | Brain_CNS | Diffuse Glioma (DIFG) | Astrocytoma (ASTR) | NA | Mesenchymal |
| SIMA | 8.099628818 | 3.333915866 | NA | Brain_CNS | Embryonal Tumor (EMBT) | Neuroblastoma (NBL) | NA | Mesenchymal |
| DBTRG-05MG | 6.472679823 | 3.309018217 | GBM | Brain_CNS | Diffuse Glioma (DIFG) | Astrocytoma (ASTR) | NA | Mesenchymal |
| SK-N-AS | 8.470759945 | 3.295022507 | NA | Brain_CNS | Embryonal Tumor (EMBT) | Neuroblastoma (NBL) | NA | Mesenchymal |
| D-336MG | 6.524727344 | 3.289991603 | OTHER | Brain_CNS | NA | NA | NA | Mesenchymal |
| NB17 | 8.848707666 | 3.26002437 | NA | Brain_CNS | Embryonal Tumor (EMBT) | Neuroblastoma (NBL) | NA | Mesenchymal |
| KP-N-YN | 9.204792365 | 3.238057675 | NA | Brain_CNS | Embryonal Tumor (EMBT) | Neuroblastoma (NBL) | NA | Mesenchymal |
| LAN-6 | 9.156733942 | 3.218550513 | NA | Brain_CNS | Embryonal Tumor (EMBT) | Neuroblastoma (NBL) | NA | Epithelial-Mesenchymal |
| D-542MG | 7.141046524 | 3.20982201 | GBM | Brain_CNS | NA | NA | NA | Mesenchymal |
| D-392MG | 8.246723978 | 3.204740961 | GBM | Brain_CNS | NA | NA | NA | Mesenchymal |
| D-247MG | 6.815748064 | 3.201227445 | GBM | Brain_CNS | NA | NA | NA | Mesenchymal |
| no-11 | 6.271905143 | 3.184216618 | OTHER | Brain_CNS | NA | NA | NA | Mesenchymal |
| DK-MG | 8.453742098 | 3.175669599 | GBM | Brain_CNS | Diffuse Glioma (DIFG) | Astrocytoma (ASTR) | NA | Mesenchymal |
| KNS-81-FD | 6.914966493 | 3.150996315 | CANNOT_CLASSIFY | Brain_CNS | Diffuse Glioma (DIFG) | Astrocytoma (ASTR) | NA | Mesenchymal |
| LN-405 | 7.761316957 | 3.136486143 | GBM | Brain_CNS | NA | NA | NA | Mesenchymal |
| BE2-M17 | 9.608514654 | 3.11526424 | OTHER | Brain_CNS | NA | NA | NA | Mesenchymal |
| CAS-1 | 7.139373664 | 3.113944637 | GBM | Brain_CNS | Diffuse Glioma (DIFG) | Astrocytoma (ASTR) | NA | Mesenchymal |
| SNB75 | 7.700290599 | 3.108134946 | GBM | Brain_CNS | NA | NA | NA | Mesenchymal |
| U-87-MG | 8.240978063 | 3.098723986 | GBM | Brain_CNS | Diffuse Glioma (DIFG) | Astrocytoma (ASTR) | NA | Mesenchymal |
| U-118-MG | 6.312173002 | 3.098642191 | GBM | Brain_CNS | Diffuse Glioma (DIFG) | Astrocytoma (ASTR) | NA | Mesenchymal |
| D-502MG | 5.936074345 | 3.011801372 | OTHER | Brain_CNS | NA | NA | NA | Mesenchymal |
