# Supplementary material for: Downregulated ferroptosis‐related gene SQLE facilitates temozolomide chemoresistance, and invasion and affects immune regulation in glioblastoma
Source: CNS Neurosci Ther. 2022 Aug 13;28(12):2104–15. doi: 10.1111/cns.13945 (PMC9627366; doi:10.1111/cns.13945)
Supplement: Supplementary file 9 — Table S7 [file CNS-28-2104-s008.docx]

**Supplementary Table S7.** The 150 differentially expressed genes that interact with SQLE from GlioVis database.

| TMSL8 | DCX | SQLE | MYT1 | NXPH1 | MEX3B | KCND2 | HES5 | RNF165 | SUSD5 |
| --- | --- | --- | --- | --- | --- | --- | --- | --- | --- |
| SYT13 | FAM77C | PAK3 | COL9A1 | COL9A3 | FLRT3 | FGFBP3 | OPCML | KIAA2022 | PAK7 |
| DLL3 | SLITRK4 | DACT2 | NMU | LOC285382 | RAB3C | TMEM132B | OVOS2 | TOX3 | KIAA1549 |
| EPHB1 | USP43 | PROM1 | GLCCI1 | DGKI | NR0B1 | SOX4 | RP11.35N6.1 | PDGFRA | MEGF11 |
| CXXC4 | HMGCS1 | PAQR9 | DOK6 | SYT4 | FHOD3 | TMEM16C | RIT2 | PLEKHK1 | MEX3A |
| ELAVL4 | MAGI1 | XKR4 | CENPF | GNG4 | LRRC4 | FBXL16 | DSCAM | KLRC3 | NEFL |
| KLRC2 | PBK | NETO1 | LUZP2 | SLITRK1 | SALL3 | SOX11 | PRR6 | IL21R | CYBA |
| CD48 | FBXO32 | GPR65 | CCL8 | LAIR1 | LY86 | CLDN23 | S100A4 | PDPN | AQP9 |
| MS4A6A | TIMP1 | CAMP | LYVE1 | CLCF1 | COPZ2 | CD33 | CXCL2 | LYZ | SPP1 |
| CD52 | CCL20 | CLEC2B | VAMP8 | OSM | ANGPTL4 | TSLP | C6orf141 | APOC2 | PI3 |
| CSTA | IFI30 | MOXD1 | GBP2 | CH25H | PTX3 | CCL7 | SRPX2 | AIF1 | SERPINA3 |
| HPR | CD69 | UBD | DKK1 | CXCL3 | BATF | FCGR2B | LOC221091 | MS4A4A | POSTN |
| CP | APOC1 | F13A1 | LGALS3 | IL6 | CXCL14 | LOX | C3 | SOD2 | C1S |
| IL8 | CCL2 | SPOCD1 | LY96 | CFD | SLPI | CFB | MMP7 | PLA2G2A | PBEF1 |
| HAMP | MYBPH | S100A9 | ABCC3 | SAA2 | NNMT | CHI3L2 | S100A8 | LTF | SAA1 |
